# Financial incentives for COVID-19 vaccines in a rural low-resource setting: a cluster-randomized trial

**DOI:** 10.1038/s41591-023-02670-4

**Published:** 2023-11-27

**Authors:** Raymond Duch, Edward Asiedu, Ryota Nakamura, Thomas Rouyard, Alberto Mayol, Adrian Barnett, Laurence Roope, Mara Violato, Dorcas Sowah, Piotr Kotlarz, Philip Clarke

**Affiliations:** 1 Nuffield College, University of Oxford, Oxford, UK; 2 Edward Asiedu and Dorcas Sowah University of Ghana Business School, University of Ghana, Accra, Ghana; 3 Hitotsubashi Institute for Advanced Study, Hitotsubashi University, Tokyo, Japan; 4 City University of New York (CUNY) Graduate School of Public Health & Health Policy, New York, NY USA; 5 Department of Public Administration, FAE University of Santiago Chile, Santiago, Chile; 6 Queensland University of Technology, Brisbane, Queensland Australia; 7 University of Oxford, Oxford, UK; 8 University of Melbourne, Melbourne, Victoria Australia

**Keywords:** Social sciences, Infectious diseases

## Abstract

We implemented a clustered randomized controlled trial with 6,963 residents in six rural Ghana districts to estimate the causal impact of financial incentives on coronavirus disease 2019 (COVID-19) vaccination uptake. Villages randomly received one of four video treatment arms: a placebo, a standard health message, a high cash incentive (60 Ghana cedis) and a low cash incentive (20 Ghana cedis). For the first co-primary outcome—COVID-19 vaccination intentions—non-vaccinated participants assigned to the cash incentive treatments had an average rate of 81% (1,733 of 2,168) compared to 71% (1,895 of 2,669) for those in the placebo treatment arm. For the other co-primary outcome of self-reported vaccinations 2 months after the initial intervention, the average rate for participants in the cash treatment was 3.5% higher than for participants in the placebo treatment (95% confidence interval (CI): 0.001, 6.9; *P* = 0.045): 40% (602 of 1,486) versus 36.3% (672 of 1,850). We also verified vaccination status of participants: in the cash treatment arm, 36.6% (355 of 1,058) of verified participants had at least one dose of the COVID-19 vaccine compared to 30.3% (439 of 1,544) for those in the placebo—a difference of 6.3% (95% CI: 2.4, 10.2; *P* = 0.001). For the intention and the vaccination outcomes, the low cash incentive (20 Ghana cedis) had a larger positive effect on COVID-19 vaccine uptake than the high cash incentive (60 Ghana cedis). Trial identifier: AEARCTR-0008775.

## Abstract (web summary)

A four-arm cluster-randomized trial testing the effects of a low cash incentive, a high cash incentive, a health message and a placebo on COVID-19 vaccine uptake in villages in rural Ghana demonstrated highest uptake with a low cash incentive.

## Main

Efforts to vaccinate populations in Africa against coronavirus disease 2019 (COVID-19) have been challenging. Despite having adequate supplies of the vaccines in many African countries, vaccination rates in Africa have remained quite low. By 4 July 2022, Africa had administered 41 COVID-19 vaccine doses per 100 population, compared to 154 for the rest of the world^1^. Cash incentives were proposed as a way to improve the efficiency and equity of the roll-out in Africa^2^. Experimental evidence suggests that financial incentives can promote certain healthcare behaviors^3,4^. Recent studies from low- and middle-income countries (LMICs) suggest that incentives increase vaccination uptake^5^. Nevertheless, a review suggests that the impact of cash incentives has been understudied in LMICs^6^.

The COVID-19 pandemic highlighted the importance of understanding whether financial incentives are an effective policy tool for promoting vaccinations^7^, particularly in African contexts. Evidence from mostly high-income countries (HICs) as to whether cash incentives promote COVID-19 vaccine uptake is mixed. For example, a randomized controlled trial (RCT) conducted in Sweden found that modest monetary payments of 24 US dollars increased vaccination rates by 4.2 percentage points^8^. A US online randomized experiment found that a $1,000 financial incentive increased the percentage of individuals who said that they would accept vaccination by 16% from a base uptake level of 70% (ref. ^9^). In contrast, an RCT involving cash incentives of up to 50 US dollars in a Medicaid managed care plan in California did not show an effect on vaccination uptake^10^. Based on their systematic review of 25 published studies of incentives for COVID-19 vaccinations in HICs, Mardi et al.^11^ concluded that high financial incentives and the Vax-a-Milion lottery resulted in higher vaccination rates, whereas low financial incentives, other lotteries and persuasive messages had small or non-significant effects. Therefore, there have been calls for a better understanding of how financial incentives shape vaccine uptake^7^.

In this context, we ran a clustered RCT with 6,963 residents in the six rural Ghana districts of Gomoa West, Asikuma Odoben Brakwa, Twifo Atti-Morkwa, Assin North, Asuogyaman and Upper Manya Krobo. The trial assesses whether financial incentives produce substantial increases in COVID-19 vaccine uptake. Our pre-registered hypothesis was that people receiving either a low or high cash incentive would have higher vaccination rates than individuals in the placebo treatment; we also expected them to have higher vaccination uptake than those viewing a standard health message related to COVID-19. An additional hypothesis was that high cash incentives would have a larger effect on vaccine uptake than low cash incentives.

We measured three outcomes: vaccination intention (immediately after treatment); reported vaccination (2 months after treatment); and verified vaccination status. Our expectation was that the hypothesized treatment effects would be observed in the case of all three outcome variables.

## Results

### Trial design

**Fig. 1:**
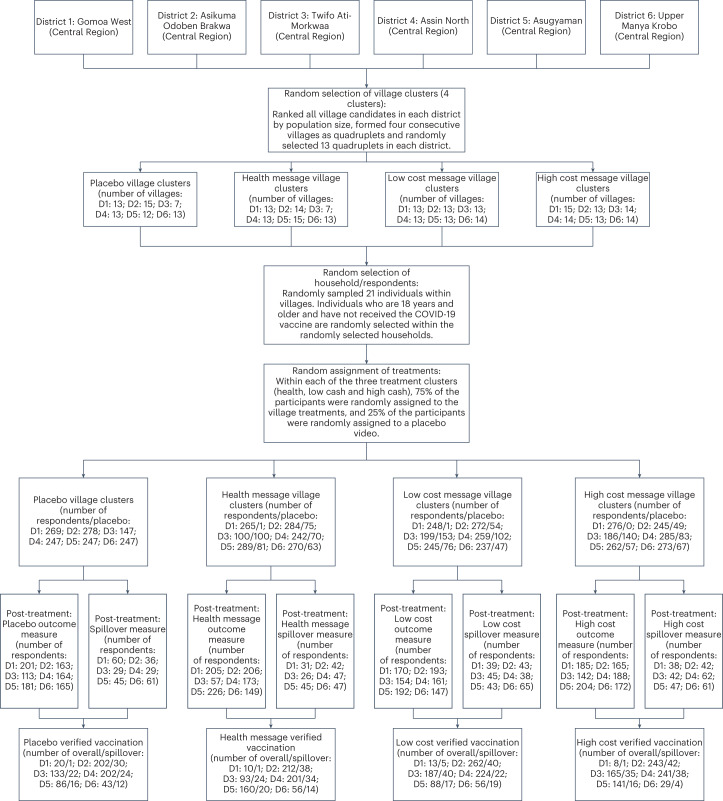
CONSORT diagram for the Ghana Financial Incentives Trial. Phase I: Treatment. Phase II/III: **a**, Post-treatment survey; **b**, Spillover Sample. Phase IV: Verification of Vaccination Status. D1 through D6 denotes districts.

The trial interventions began on 5 February 2022. The CONSORT diagram (Fig. 1) describes the random allocation of 310 village clusters to one of the four treatment arms. In each of the six districts, our District Health Office partners identified villages that could be feasibly enumerated (the primary consideration here was the quality of the road access); villages were ranked according to their population size; and each four consecutive villages were designated a quadruplet. A typical district had approximately 50 quadruplets. In each district, we randomly selected 13 quadruplets, with probabilities weighted by the quadruplet’s share of the total population of the district’s villages. Within each quadruplet, villages were randomly assigned one of four video treatment arms: a placebo, a standard health message, a high cash incentive (60 Ghana cedis/$10) and a low cash incentive (20 Ghana cedis/$3). A total of 310 villages were included, with about 77 villages assigned to each of the four treatment arms. The population size of the sampled villages in the trial varied between 30 and 5,428, with a median size of 1,040. Within each village, we randomly selected 21 households, and, within households, we randomly selected a single eligible individual (18 years of age or older). This resulted in a baseline sample of 5,900 participants. Our pre-registered power analyses indicated that we would be powered at 0.80 to detect an effect size of 0.06 with four treatment arms, 310 villages and 21 treated individuals per village.

This is a two-stage randomized trial in which the treatment regimen is defined such that at least one individual in a treated cluster receives treatment, and, in control clusters, all individuals are allocated to control^12^. Given that a village cluster was assigned one of the non-placebo treatment videos (health, low cash or high cash), 75% of randomly selected participants saw the village-assigned treatment; 25% of the participants were randomly assigned the placebo treatment. We included these placebo treatments to facilitate the identification of cluster-level spillover effects (or indirect effects) that compare individuals assigned the placebo video in treated clusters with individuals receiving the placebo video in a placebo cluster^13–15^. During the period 5–28 February 2022, a total of 5,900 individuals were treated: 2,669 with the placebo; 1,063 with the health message; 1,079 with the low financial incentive (20 Ghana cedis/$3); and 1,089 with the high financial incentive (60 Ghana cedis/$10).

Beginning on 13 April 2022, 2 months after the initial intervention, participants were contacted by telephone and asked a short survey including a question about their vaccination status (the survey is available in the Supplementary Information). In this phase II, a total of 2,082 individuals, from the 5,900 originally treated individuals, were successfully contacted by telephone (a response rate of approximately 35%).

Phase III began on 15 June 2022 with two components. In this Reported Vaccination component, enumerators contacted all participants who were not successfully contacted by telephone in phase II of the trial. The 2,019 participants successfully contacted in phase III were asked an identical set of questions to those included in the phase II post-treatment survey.

Phase III also entailed an Enumeration of a Sample of Spillover Households consisting of non-treated individuals in treated villages. The spillover sample was drawn from approximately 80 villages in each of the six districts. After enumerators finished surveying each of their recontacted households, they tossed a fair coin to determine whether they would then randomly sample a spillover household. From our treated villages, we obtained a sample of 1,101 non-treated households; these individuals answered the phase III spillover survey (Supplementary Information).

Between 15 October and 30 November 2022, in phase IV, Verified Vaccine Status, the staff of the six District Health Offices were supplied with a list of the 5,900 phase I participants and the 1,101 participants in the spillover sample. The District Health Offices returned a list indicating whether, and when, each of the trial participants had received at least one dose of a COVID-19 vaccine.

### Covariates

Summary mean and standard deviation statistics for the phase I sample are presented in Table 1. The sample included slightly more females (gender is self-reported by respondents to the survey); the average participant age was 37 years; average household size was five with, on average, slightly less than two individuals under the age of 18 years; approximately 12% of participants were unemployed; average weekly spending on food was about 160 Ghana cedis (approximately $20); weekly non-food spending averaged about 37 cedis; and 18% of participants never attended school, whereas 62% attended middle school or higher. Average distance (in kilometers) to a health clinic is our proxy for access to the COVID-19 vaccine; the mean overall value is 5.4 km, with some variation across treatment arms. Respondents scored an average of 2.3 on a five-point scale (from very bad to very good) rating the overall economic or financial condition of their household (average finances score).

**Table 1:** Summary statistics for survey results from phase I

	Total sample	Placebo	Health message	Low cash	High cash
Sample					
Number of villages	310	73	75	79	83
Number of participants	5,900	2,669	1,063	1,079	1,089
Treatment outcomes					
Vaccine intention	74.6 (43.5)	71.0 (45.4)	72.5 (44.7)	81.7 (38.7)	78.2 (41.3)
Certainty of vaccination	63 (30.4)	60.7 (31.2)	62.1 (30.9)	67.9 (27.6)	64.9 (30.2)
Solar device intention	3.9 (19.3)	4.8 (21.4)	2.2 (14.6)	4.5 (20.8)	2.7 (16.2)
Certainty of solar intention	48.0 (32.8)	51.7 (32.3)	43.9 (33.2)	47.8 (32.3)	43.1 (33.1)
Covariates					
Female (%)	57.1 (49.5)	54.1 (49.8)	57.9 (49.4)	58.6 (49.3)	61.9 (48.6)
Mean age	37.4 (16.4)	37.3 (16.1)	37 (16.9)	36.9 (16.1)	38.4 (16.8)
Mean household size	5 (2.7)	5 (2.5)	5 (3.0)	5 (2.7)	5.1 (2.7)
Mean number of children *<*18 years old	1.8 (2.2)	1.7 (1.6)	1.9 (2.0)	1.9 (1.7)	1.9 (3.5)
Employed full time (%)	59.0 (49.2)	60.8 (48.8)	56.6 (49.6)	57.5 (49.5)	58.6 (49.3)
Employed part time (%)	13.7 (34.4)	14.4 (35.1)	14.2 (34.9)	13.2 (33.8)	12.1 (32.7)
Unemployed (%)	12.6 (33.2)	11.5 (31.9)	12.5 (33.1)	15.6 (36.3)	12.7 (33.3)
Mean weekly spend food	161.6 (99.7)	161.9 (102.1)	159.3 (95.1)	161.3 (98.4)	163.5 (99.8)
Mean weekly spend non-food	37.1 (52.1)	34.5 (46)	36.1 (49.1)	39.7 (54.6)	41.7 (64.9)
Average finances score	2.3 (1)	2.4 (1.1)	2.3 (1)	2.3 (1)	2.4 (1)
Never attended school (%)	18.1 (38.5)	16.6 (37.2)	17.9 (38.3)	19 (39.2)	21.2 (40.9)
Middle school or higher (%)	62.3 (48.5)	63.9 (48)	63.7 (48.1)	59.8 (49.1)	59.5 (49.1)
Distance to clinic in kilometers	5.6 (4.4)	5.7 (4.5)	5.2 (3.7)	4.7 (3.7)	6.5 (5)

Note: Mean values are presented for treatment outcomes and covariates. Their standard deviations are presented in parentheses.

### Vaccine intentions

Immediately after receiving the video information treatment, 75% of participants signaled their intention to get vaccinated (with an intracluster correlation coefficient of 0.15 (95% confidence interval (CI): 0.13, 0.18)). The placebo arm had the lowest vaccine intention rate of 71.0%. The health message arm had a slightly higher rate of 72.5% (difference from placebo: 1.5; 95% CI: −1.7, 4.7; *P* = 0.37). Throughout, we report two-sided *t*-tests for the difference in means. The high cash incentive arm registered 78.2% (difference from placebo: 7.2; 95% CI: 4.0, 10.4; *P* < 0.001). The low cash incentive expressed the highest intention to get vaccinated of 81.7% (difference from placebo: 10.7; 95% CI: 7.5, 13.8; *P* < 0.0001). Vaccine intentions of individuals in low cash were higher than for those in high cash (low cash difference from high cash: 3.5; 95% CI: 0.07, 7.0; *P* = 0.048).

### Reported vaccinations

**Extended Data Fig. 1:**
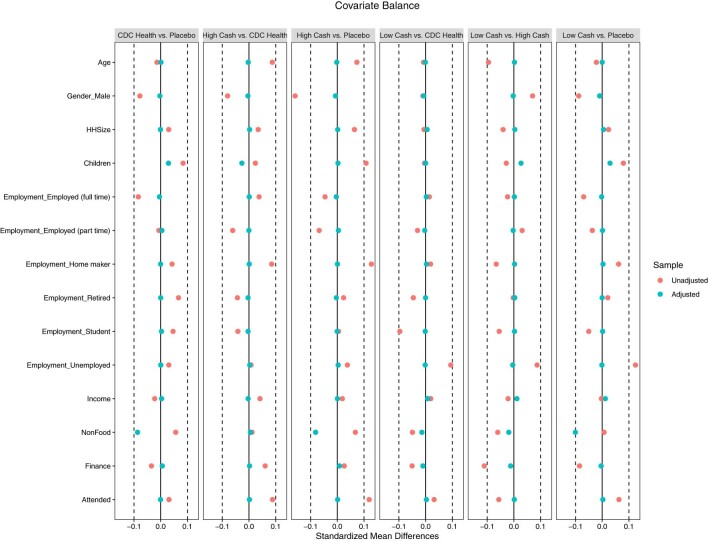
**Balance on Standardized Mean Differences for Full Phase I Sample: Unadjusted compared to propensity score weighted sample**.

Self-reported vaccination status and other post-treatment outcomes and covariates are presented in Table 2. The first set of outcomes was collected either by telephone survey in phase II or in-person in phase III. A total of 4,101 participants were recontacted from 303 villages. This represented an attrition rate of about 30%. Extended Data Table 1 presents these descriptive results disaggregated by phase II (cell phone) and phase III (in-person) recontacted participants. We conducted extensive analyses of sample attrition, reported in the Methods and also in Extended Data Fig. 1 and Extended Data Tables 2–4, all of which suggested that attrition did not bias the estimated treatment effects. Participants were asked whether, and when, they had received at least one dose of the COVID-19 vaccine. Thirty-eight percent of this recontacted sample reported having received the COVID-19 vaccine (with an intracluster correlation coefficient of 0.06 (95% CI: 0.05, 0.09)). The placebo condition had the lowest reported vaccination rate of 36.4%. The health message arm had no significant difference in rates (difference from placebo: 2.1; 95% CI: −2.0, 6.2; *P* = 0.31). The high cash incentive arm registered 38.1% (difference from placebo: 1.7; 95% CI: −2.3, 5.9; *P* = 0.41). The low cash arm reported the highest vaccination rate of 41.8% (difference from placebo: 5.4; 95% CI: 1.4, 9.6; *P* = 0.009). Reported vaccinations in the low cash treatment were higher than for those in the high cash treatment (low cash difference from high cash: 3.7; 95% CI: −1.2, 8.7; *P* = 0.14). There are differences across data collection modes: phase II reported vaccination rates (44%) are higher than those for phase III (33%); in phase II, low cash had the highest reported vaccination rate of 48.1% (difference from placebo: 7.6; 95% CI: 1.7, 13.4; *P* = 0.012), and although lower than phase II, low cash also had the highest rate of 35.0% in phase III (difference from placebo: 2.9; 95% CI: −0.029, 0.087; *P* = 0.33).

**Table 2:** Summary statistics for phase II and III (post-treatment telephone and in-person interviews: reported vaccinations) and phase IV (verified vaccination status)

	Total sample	Placebo	Health message	Low cash	High cash
Phase II and III: telephone and in-person post-treatment participants interview					
Number of villages	303	72	73	77	81
Number of participants	4,101	1,850	765	747	739
Female (%)	56.0 (49.6)	52.8 (49.9)	57.9 (49.4)	58.4 (49.3)	59.7 (49.1)
Reported vaccinated (%)	38.1 (48.6)	36.3 (48.1)	38.5 (48.7)	41.9 (49.4)	38.1 (48.6)
Mean villages visited last month	1.8 (1.9)	1.8 (1.9)	1.8 (1.6)	1.9 (1.9)	1.9 (2.2)
Mean villages visited last year	4.9 (6.2)	4.9 (5.6)	5.1 (6.8)	5 (6.7)	4.9 (6.4)
Percent with family in other villages	70.9 (0.5)	69.9 (45.7)	70.8 (45.3)	71.2 (44.9)	72.9 (44.2)
Percent with WhatsApp	28.4 (45.1)	27.7 (44.8)	30.2 (46)	30.1 (46)	26.4 (44.2)
Percent using WhatsApp ≥ once per month	98.8 (10.9)	99.0 (9.9)	99.6 (6.6)	97.4 (16)	99 (10.1)
Phase IV: verified vaccination status of treated participants (30 April 2022)					
Number of villages	234	52	58	62	62
Number of participants	3075	1544	473	548	510
Female (%)	55.7 (49.7)	54.1 (49.8)	55.8 (49.7)	56.4 (49.6)	59.6 (49.1)
Actual vaccination (%)	29.1 (45.4)	28.4 (45.1)	21.6 (41.2)	40.7 (49.2)	25.9 (43.8)
Mean villages visited last month	1.9 (1.9)	1.8 (2)	1.7 (1.6)	1.9 (1.8)	2 (1.9)
Mean villages visited last year	5 (6.4)	4.9 (6.1)	4.8 (5.6)	5.1 (5.7)	5.3 (8.3)
Percent with family in other villages	49.9 (45.8)	49.8 (46)	49.3 (47.2)	49.1 (46.2)	51.8 (42.6)
Percent with WhatsApp	20.9 (45.6)	21 (45.6)	25.2 (47.5)	19.7 (44.8)	18 (44.2)
Percent using WhatsApp ≥ once per month	98.5 (12.4)	98.8 (11.1)	100 (0)	96.3 (18.9)	97.9 (14.5)

Note: Mean values are presented for treatment outcomes and covariates. Their standard deviations are presented in parentheses.

### Verified vaccination status

In phase IV, District Health Office officials confirmed the vaccination status of 3,075 trial participants. This represents about 50% of the pre-treatment sample of 5,900. The Methods section and Extended Data Tables 3 and 4 present extensive analyses of sample attrition that indicate that attrition did not bias the estimated treatment effects.

The lower panel of Table 2 reports the vaccination status of individuals in the four treatment arms as of the end of April 2022, which is 2 months after the trial began implementing the video treatments. For our six-district sample, we verified that 29.1% of the 3,075 participants received a COVID-19 vaccination (almost identical to the 30% May 2022 national rate reported by Our World in Data^16^). The intracluster correlation coefficient for verified vaccinations is 0.64 (95% CI: 0.60, 0.69). We verified that 28.4% of individuals in the placebo arm were vaccinated (at least one dose); 21.6% of individuals in the health message arm were vaccinated (at least one dose) (difference from placebo: −6.8; 95% CI: −11.4, −2.3; *P* = 0.003); 40.7% of individuals in the low cash arm were vaccinated (at least one dose) (difference from placebo: 12.3; 95% CI: 7.8, 16.8; *P* < 0.0001); and 25.9% of individuals in the high cash treatment were vaccinated (at least one dose) (difference from placebo: −2.5; 95% CI: −7.0, 1.9; *P* = 0.27). This is strong evidence that the low cash financial incentives had a significant positive effect on vaccination uptake, but the high cash financial incentives had no significant effect, and the health treatment depressed vaccination uptake compared to the placebo.

### Combined low and high cash treatment effect

Our pre-registered hypothesis was that the average vaccination rates for those in the cash treatments (low and high) would be higher than those in the placebo and higher than those in the health message treatments.

Vaccine intentions were positively affected by the cash treatment. Individuals in a cash treatment arm (low or high cash) had an average vaccine intention rate of 80.0% compared to 71.0% for individuals in the placebo treatment arm (difference from placebo: 9.0%; 95% CI: 6.4, 11.4; *P* < 0.0001) and also higher than the 72.5% in the health message treatment arm (difference from health message: 7.5%; 95% CI: 4.3, 10.5; *P* < 0.0001).

Cash also increased reported vaccination rates. Thirty-eight percent of participants surveyed 8 weeks after the intervention indicated that they had been vaccinated. In the cash treatment arm, 40% of participants reported having had a COVID-19 vaccine since the phase I intervention (only non-vaccinated individuals were eligible for the phase I intervention). Participants in the cash treatment arm had higher reported vaccination rates than the 36.3% for those in the placebo treatment arm (difference from placebo: 3.7%; 95% CI: 0.3, 6.9; *P* = 0.028) and also somewhat higher than those in the health message treatment arm (difference from health message: 1.5%; 95% CI: −5.8, 2.8; *P* = 0.49). The 3.7% cash effect on reported vaccination status represents about a 10% increase over the non-treated placebo rates.

Similarly, combined cash treatment effects were observed for verified vaccination rates. In the cash treatment arm (both low and high cash), we verified that 33.6% of individuals had at least one dose of the COVID-19 vaccine as of April 2023 compared to 28.4% of those in the placebo arm (difference from placebo: 5.2%; 95% CI: 1.5, 8.7; *P* = 0.005). Individuals in the cash arm also had higher verified vaccination rates than those in the health message treatment arm (difference from health message: 9.8%; 95% CI: 7.1, 16.9; *P* < 0.0001). In relative terms, this represents about an 18% increase over the non-treated placebo vaccination rates.

### Multiple variable models

Table 3 reports the odds ratio results for multiple variable models, including treatment effects and covariates. Results are reported for each of the three outcome measures: intentions, reported vaccine status and verified vaccination. Model 1 reports odds ratios for the logistic regression of vaccine intentions (measured immediately after the phase I intervention) on dummy variables indicating whether participants received one of the cash incentive videos (Cash) or the health message (Health); the estimation includes the full set of covariates (access to a health clinic, age, education, gender, employment status, average weekly food expenditures and the density of social media network). We also include district fixed effects. The results are consistent with our pre-registered conjecture: the odds of participants in the cash video treatments expressing an intention to get the COVID-19 vaccine are about 1.72 times the odds for the placebo participants. Distance from the nearest health clinic has little impact on vaccination intention. Age is negatively correlated with the intention to get the COVID-19 vaccine. Low-educated participants are more likely to express an intention. The second intention model (model 2) includes the low cash and high cash treatments as separate dummy variables. The low cash treatment has a strong effect: the intention of these treated participants to get the COVID-19 vaccine is almost twice the odds for the participants in the placebo arm.

**Table 3:** Odds ratios and 95% CIs from regression of vaccine outcomes on treatments and covariates

	Intention	Intention	Reported	Reported	Actual	Actual
Cash	1.79 (1.45, 2.22)		1.21 (1.02, 1.45)		1.47 (0.92, 2.36)	
High cash		1.69 (1.27, 2.25)		1.15 (0.91, 1.45)		0.97 (0.45, 2.09)
Low cash		1.91 (1.44, 2.54)		1.28 (1.04, 1.57)		2.00 (1.13, 3.54)
Health	1.15 (0.87, 1.51)	1.15 (0.87, 1.51)	1.09 (0.89, 1.34)	1.09 (0.89, 1.33)	0.70 (0.34, 1.42)	0.69 (0.34, 1.40)
Access	1.04 (0.89, 1.21)	1.05 (0.90, 1.22)	0.95 (0.86, 1.06)	0.96 (0.86, 1.07)	1.18 (0.78, 1.80)	1.24 (0.81, 1.87)
Age (+10 years)	0.83 (0.79, 0.87)	0.83 (0.79, 0.87)	0.96 (0.92, 1.01)	0.96 (0.92, 1.01)	1.03 (0.95, 1.13)	1.04 (0.95, 1.13)
Male	1.10 (0.97, 1.26)	1.10 (0.96, 1.25)	0.87 (0.76, 1.00)	0.87 (0.76, 1.00)	0.97 (0.75, 1.27)	0.97 (0.75, 1.27)
High-educated	0.68 (0.40, 1.17)	0.69 (0.40, 1.17)	1.15 (0.69, 1.91)	1.15 (0.69, 1.92)	1.85 (0.77, 4.46)	1.98 (0.82, 4.75)
Medium-educated	0.99 (0.79, 1.23)	0.99 (0.79, 1.23)	1.15 (0.90, 1.48)	1.16 (0.90, 1.49)	1.78 (1.18, 2.71)	1.84 (1.22, 2.76)
Low-educated	1.26 (1.06, 1.51)	1.26 (1.06, 1.51)	1.01 (0.80, 1.27)	1.01 (0.80, 1.27)	1.27 (0.87, 1.86)	1.34 (0.93, 1.93)
Employed	0.91 (0.79, 1.06)	0.91 (0.79, 1.06)	0.91 (0.79, 1.05)	0.91 (0.79, 1.05)	1.14 (0.89, 1.47)	1.14 (0.88, 1.46)
Mean food (+50)	0.94 (0.90, 0.98)	0.94 (0.90, 0.98)	1.00 (0.97, 1.04)	1.01 (0.97, 1.04)	1.03 (0.95, 1.13)	1.03 (0.95, 1.12)
Social media (+10)			1.09 (0.98, 1.22)	1.09 (0.98, 1.22)	1.06 (0.91, 1.23)	1.06 (0.91, 1.24)
District 2	1.62 (1.10, 2.39)	1.62 (1.10, 2.38)	1.31 (1.02, 1.68)	1.31 (1.02, 1.67)	0.03 (0.01, 0.10)	0.03 (0.01, 0.10)
District 3	1.30 (0.79, 2.13)	1.29 (0.78, 2.11)	1.05 (0.79, 1.41)	1.04 (0.78, 1.39)	0.04 (0.02, 0.11)	0.04 (0.02, 0.11)
District 4	0.76 (0.53, 1.09)	0.76 (0.53, 1.09)	1.07 (0.82, 1.40)	1.07 (0.82, 1.40)	0.11 (0.04, 0.31)	0.11 (0.04, 0.32)
District 5	0.69 (0.48, 1.01)	0.70 (0.48, 1.01)	0.66 (0.52, 0.83)	0.66 (0.52, 0.83)	0.01 (0.00, 0.02)	0.01 (0.00, 0.02)
District 6	0.44 (0.30, 0.65)	0.44 (0.30, 0.65)	0.50 (0.36, 0.69)	0.50 (0.36, 0.68)	0.15 (0.05, 0.41)	0.14 (0.05, 0.40)
Observations	5,644	5,644	3,957	3,957	2,146	2,146
AIC	6,033	6,034	5,173	5,174	2,242	2,228
log likelihood	−3,001	−3,000	−2,569	−2,569	−1,104	−1,096

Note: 95% CIs are reported in parentheses. The CIs are based on standard errors that are clustered at the village level. AIC, Akaike information criterion.

The second set of models presents similar results for reported vaccine status that was obtained, 2 months after individuals were treated, either by telephone in phase II of the trial or by the phase III in-person follow-up. Reported vaccination status in phase II and phase III (model 3) was considerably lower than intention levels, hence the lower intercept term. In post-treatment, the odds of individuals in the cash treatment reporting having a COVID-19 vaccine were about 1.21 times greater than the odds for those in the placebo arm. For the model specification with both low and high cash treatments (model 4), low cash participants have 1.28 times the odds of those in placebo reporting a COVID-19 vaccination. The covariate controls have little effect on reported vaccinations; the exception is that respondents with denser social networks are more likely to report having received the vaccine.

The final set of models in Table 3 presents similar odds ratio results for those having verified vaccinations as of 30 April 2022. The estimated odds of verifying COVID-19 vaccinations for individuals in the combined cash treatments (model 5) were about 1.5 times the estimated odds of individuals in the placebo treatment, although the confidence intervals include 1.0. In the model including both low and high cash treatments (model 6), the low cash odds ratio is 2.0 and is statistically significant. Verified COVID-19 vaccination rates were positively correlated with age and were higher for those with medium education.

**Extended Data Fig. 2:**
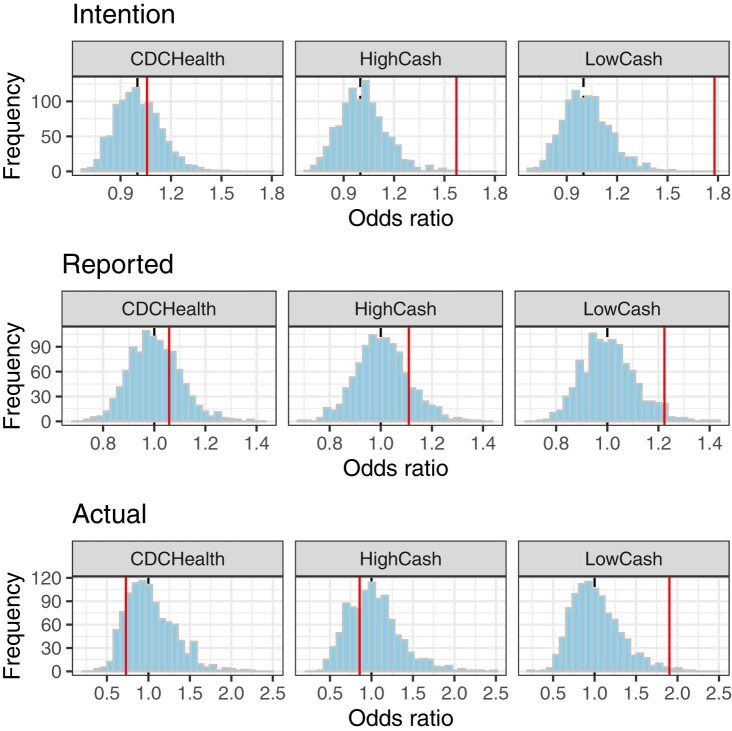
**Randomization Inference Simulated Odds Ratios for Models in Table** 3. **(Vertical red line indicating observed odds ratio)**.

Extended Data Fig. 2 presents the bootstrapped simulations for calculating randomization inference (RI) *P* values for the treatment effect estimations in Table 3; the RI *P* values are very similar to those presented in Table 3 (Supplementary Table 3 compares the *P* values).

**Fig. 2:**
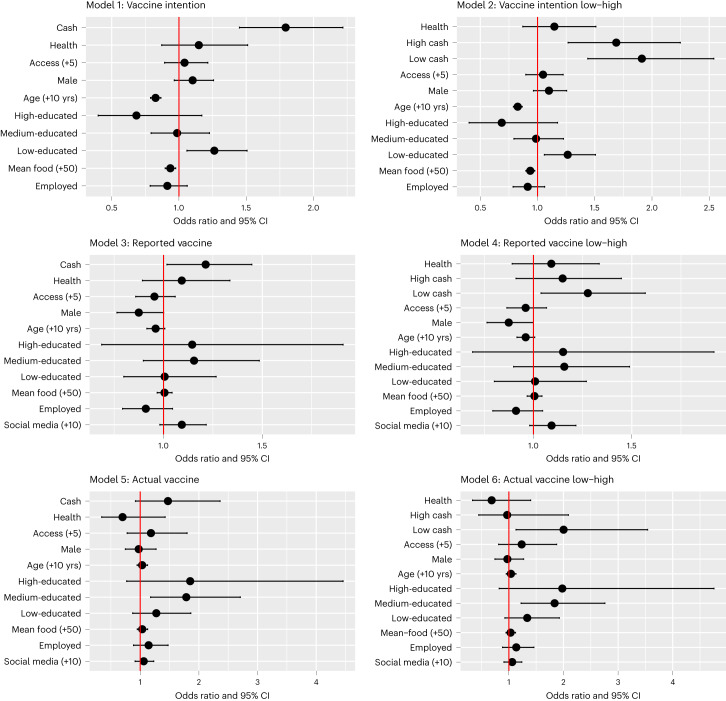
Financial incentive and vaccination outcomes. Odds ratios for model estimates in Table 3. Odds ratios are reported for the logistic regressions for the three outcome variables: vaccine intention (models 1 and 2 in Table 3), reported vaccination (models 3 and 4 in Table 3) and verified vaccination (models 5 and 6 in Table 3). These outcomes are regressed on dummy variables indicating whether individuals received one of the cash incentive videos (Cash) or the health message (Health); the estimation includes the full set of covariates. The 95% CIs are reported for each odds ratio. Observations are in parentheses: model 1 (5,644); model 2 (5,644); model 3 (3,957); model 4 (3,957); model 5 (2,146); and model 6 (2,146).

Figure 2 summarizes the odds ratios for the vaccination outcomes. Across the three outcomes—intended, reported and verified vaccinations—low cash has the largest, and consistently statistically significant, treatment effect. High cash is statistically significant only in the intention model. Most striking is that the odds of verified COVID-19 vaccinations for individuals in the low cash arm are about double those of individuals in the placebo treatment arm.

### Spillover

A second pre-registered hypothesis is that proximity to households treated with a cash incentive treatment would depress COVID-19 vaccination uptake. The trial design allows us to test this proposition. First, within each non-placebo-treated village cluster, the two-stage random cluster design assigns 25% of individuals to the placebo treatment. For each of the three treatments, we can estimate a cluster-level spillover effect^12^.

The ‘depression’ spillover conjecture implies that, on average, the participants receiving a placebo video in cash treatment villages will have lower vaccination rates than participants in placebo-treated villages. Panel A of Extended Data Table 5 reports the spillover estimates for reported vaccinations measured in phase II and phase III surveys. Placebo participants in placebo-treated village clusters had an average reported vaccination rate of 36.3%. Placebo participants in high cash treatment villages had an average reported vaccination rate of 38.0% (difference from placebo: 1.8%; 95% CI: −4.5, 8.0; *P* = 0.58). Placebo participants in low cash treatment villages had an average reported vaccination rate of 38% (difference from placebo: 1.7%; 95% CI: −4.5, 7.9; *P* = 0.59). Placebo participants in health treatment villages had an average reported vaccination rate of 32.8% (difference from placebo: −5.2%; 95% CI: −9.6, 3.1; *P* = 0.30). We observed little evidence of negative spillover effects in reported vaccination rates. In fact, the average low cash spillover effect is positive with narrow CIs, suggesting that spillover could range between maximum negative effects of about −5.0% to maximum positive effects of about 8.0%.

Extended Data Table 5 presents similar results for verified vaccinations from phase IV. Participants in placebo-treated village clusters had an average vaccination rate of 26.8%. Participants receiving the placebo video in high cash treatment villages had an almost identical verified vaccination rate of 26.3% (difference from placebo: 0.5%; 95% CI: −6.7, 5.6; *P* = 0.86). Individuals receiving the placebo video in low cash treatment villages had a verified vaccination rate of 38.3% (difference from placebo: 11.5%; 95% CI: 5.2, 17.8; *P* < 0.001). Placebo participants in health treatment villages had an average reported vaccination rate of 22.8% (difference from placebo: −4.0%; 95% CI: −10.2, 2.1; *P* = 0.20). Placebo participants in the combined cash treatment also had a verified vaccination rate, 36.6%, that is significantly larger than the placebo treatment village participants (difference from placebo: 6.3%; 95% CI: 2.4, 10.2; *P* = 0.002). Average effects of verified vaccination rates for the spillover from low and high cash treatments are positive. Moreover, in the case of the low cash treatment, we report a mean difference estimate that ranges between 7% and 21%. To the extent that spillover effects from financial incentives exist, they tend to raise the vaccination rates of the non-incentivized rather than depress them.

Our second approach to measuring spillover is to survey, in phase III, individuals who received no video interventions. The ‘depression’ spillover conjecture implies that the average reported vaccination rates of non-treated individuals in cash-treated villages will be significantly lower than for those non-treated individuals in placebo-treated villages. Extended Data Table 5 also presents the reported vaccination rates for the phase III spillover sample. The average reported dose 1 vaccination rate for the non-treated sample was 55.5%. The non-treated in the placebo condition had a reported vaccination rate (at least one dose) of 53.9%. The non-treated in the health message arm had a reported rate of 53.8% (difference from placebo: −0.1; 95% CI: −8.9, 8.7; *P* = 0.99). The non-treated in the high cash incentive arm registered 58.3% (difference from placebo: 4.4; 95% CI: −3.9, 12.7; *P* = 0.30), and the non-treated in the low cash arm reported a vaccination rate of 55.5% (difference from placebo: 1.6; 95% CI: −6.9, 10.2; *P* = 0.71).

Extended Data Table 5 reports average verified vaccination rates for the 515 individuals in the non-treated spillover sample. Non-treated individuals in placebo-treated village clusters had a verified vaccination rate of 29.8%. Non-treated individuals in the health message arm had a verified rate of 19.6% (difference from placebo: −10.2; 95% CI: −23.2, 2.6; *P* = 0.12). Non-treated individuals in the high cash incentive arm registered 31.9% (difference from placebo: 2.1; 95% CI: −11.5, 15.5; *P* = 0.77). The low cash arm verified vaccination rate for non-treated individuals was 38.3% (difference from placebo: 8.5; 95% CI: −5.6, 22.5; *P* = 0.24).

We surveyed a randomly selected non-treated sample in the post-treatment phase III explicitly to measure spillover effects from the two cash treatments. The results are consistent with those reported for the placebo spillover estimations. There is no evidence that financial incentives to treated individuals depressed the non-treated individuals in the same village. In fact, there is some evidence to suggest that the verified vaccination rates of the non-treated individuals in the low cash treatment arms were positively affected by their proximity to individuals receiving financial incentives for the COVID-19 vaccine. We are sufficiently powered to conclude that this effect likely ranges between about 3% and 20%.

The four multi-variable models in Table 4 are based on the data summarized in Extended Data Table 5. The first two models in Table 4, reported and verified vaccination, include dummy variables for individuals receiving placebo videos in the three treated villages (HealthPlacebo, LowCashPlacebo and HighCashPlacebo). For the reported vaccination model, LowCashPlacebo has an odds ratio of 1.01 (with a CI of 0.73, 1.41), and HighCashPlacebo has an odds ratio of 1.07 (CI 0.75, 1.51). For verifed vaccinations, LowCashPlacebo has an odds ratio of 1.83 (with a CI of 0.83, 4.03), and HighCashPlacebo has an odds ratio of 1.32 (CI 0.57, 3.06). The last two models include VCash.Health, VCash.HighCash and VCash.LowCash dummy variables identifying the non-treated individuals in, respectively, the health, high cash and low cash treated villages. In model 3 for reported vaccinations, VCash.LowCash has an odds ratio of 0.98 (with a CI of 0.61, 1.59), and VCash.HighCash has an odds ratio of 1.26 (CI 0.80, 1.97). For the last model, verifed vaccinations, VCash.LowCash has an odds ratio of 1.60 (with a CI of 0.60, 4.27), and VCash.HighCash has an odds ratio of 1.22 (CI 0.48, 3.11). We found no evidence of spillover effects in the four models: the LowCashPlacebo, HighCashPlacebo, VCash.LowCash and VCash.HighCash variables have odds ratios with wide CIs that include 1.0.

**Table 4:** Spillover samples: odds ratios from regression of vaccine outcomes on treatments and covariates

	Vaccine reported	Actual vaccine	Vaccine reported SP	Actual vaccine SP
Health	1.08 (0.85, 1.36)	0.83 (0.34, 2.04)		
HighCash	1.12 (0.87, 1.43)	1.26 (0.51, 3.13)		
LowCash	1.29 (1.01, 1.64)	2.40 (1.08, 5.36)		
Health_Placebo	0.83 (0.56, 1.23)	1.07 (0.46, 2.46)		
HighCash_Placebo	1.07 (0.75, 1.51)	1.32 (0.57, 3.06)		
LowCash_Placebo	1.01 (0.73, 1.41)	1.83 (0.83, 4.03)		
VCash_Health			0.94 (0.61, 1.46)	0.58 (0.22, 1.55)
VCash_HighCash			1.26 (0.80, 1.97)	1.22 (0.48, 3.11)
VCash_LowCash			0.98 (0.61, 1.59)	1.60 (0.60, 4.27)
Access (+5)	0.96 (0.86, 1.07)	1.19 (0.78, 1.83)	0.93 (0.90, 0.95)	0.98 (0.93, 1.04)
Age (+10 years)	0.96 (0.92, 1.01)	1.03 (0.95, 1.13)	1.22 (1.08, 1.37)	0.98 (0.81, 1.18)
Male	0.87 (0.76, 1.00)	0.97 (0.75, 1.27)	0.55 (0.40, 0.75)	1.03 (0.66, 1.61)
High-educated	1.17 (0.70, 1.94)	1.93 (0.78, 4.79)	2.12 (0.69, 6.52)	2.86 (0.57, 14.47)
Medium-educated	1.16 (0.90, 1.49)	1.85 (1.23, 2.78)	2.77 (1.58, 4.84)	2.49 (0.93, 6.63)
Low-educated	1.01 (0.80, 1.27)	1.35 (0.93, 1.95)	1.51 (0.91, 2.49)	1.70 (0.66, 4.38)
Employed	0.91 (0.79, 1.05)	1.12 (0.87, 1.44)	0.93 (0.68, 1.26)	0.79 (0.49, 1.27)
Mean food (+50)	1.01 (0.97, 1.04)	1.03 (0.95, 1.12)	1.17 (1.08, 1.26)	1.06 (0.96, 1.17)
Social media (+10)	1.09 (0.98, 1.22)	1.06 (0.91, 1.24)	1.50 (1.01, 2.24)	1.09 (0.65, 1.83)
District 2	1.31 (1.02, 1.69)	0.03 (0.01, 0.09)	1.33 (0.79, 2.24)	0.14 (0.01, 1.99)
District 3	1.05 (0.78, 1.40)	0.04 (0.01, 0.10)	1.57 (0.85, 2.90)	0.10 (0.01, 1.30)
District 4	1.08 (0.82, 1.42)	0.10 (0.03, 0.30)	2.23 (1.29, 3.84)	0.44 (0.03, 5.98)
District 5	0.66 (0.52, 0.84)	0.01 (0.00, 0.02)	1.18 (0.71, 1.94)	0.01 (0.00, 0.32)
District 6	0.50 (0.36, 0.69)	0.13 (0.05, 0.38)	0.82 (0.50, 1.36)	0.33 (0.02, 4.49)
Intercept	0.70 (0.52, 0.95)	4.03 (1.49, 10.92)	0.82 (0.43, 1.56)	1.32 (0.08, 22.56)
Observations	3,957	2,146	1,035	499
AIC	5,177	2,227	1,305	548
log likelihood	−2,567	−1,092	−634	−256

Note: 95% CIs are reported in parentheses. The CIs are based on standard errors that are clustered at the village level. AIC, Akaike information criterion; SP, spillover.

One possible mechanism that facilitates spillover is the proximity of village residents treated with financial incentives to those who were not. We might expect higher levels of spillover in smaller villages where news of cash incentives spread more easily. This suggests that the effect sizes of our spillover metrics would vary by the village’s population size. We interact village population with both sets of spillover metrics: (1) VCash.Health, VCash.HighCash and the VCash.LowCash and (2) VCash.HighCash and VCash.LowCash. Results are reported in Supplementary Table 5. In the case of both sets of spillover metrics, the interaction terms are statistically insignificant. There is no evidence that spillover effects vary by village population size.

### Treatment effect heterogeneity

**Extended Data Fig. 3:**
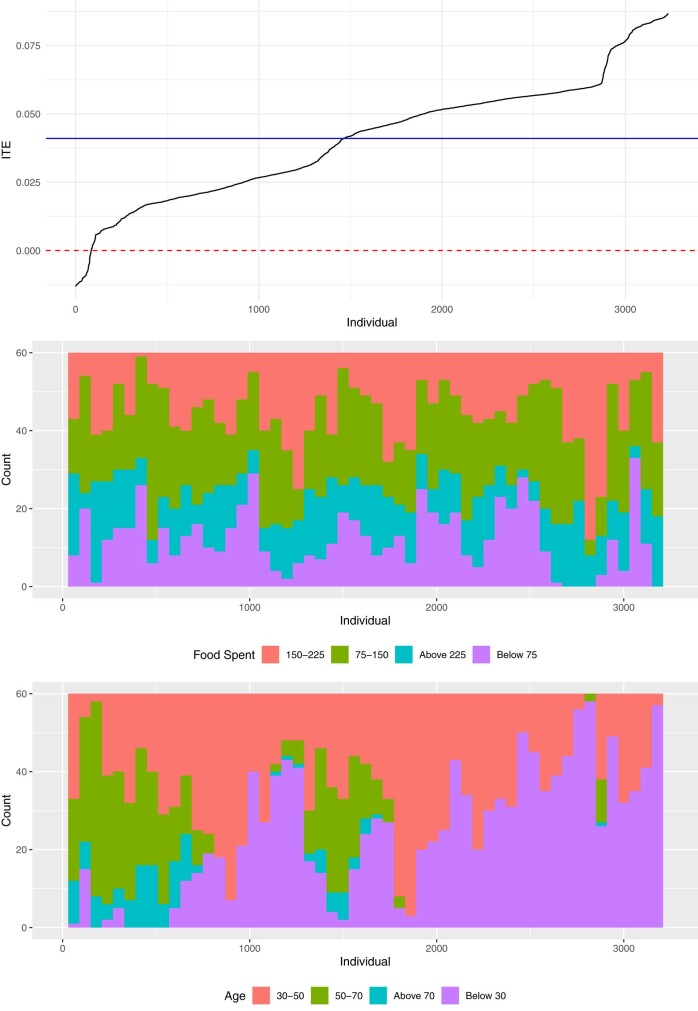
**BART: Heterogeneous Treatment Effects (Food Spending and Age)**.

**Extended Data Fig. 4:**
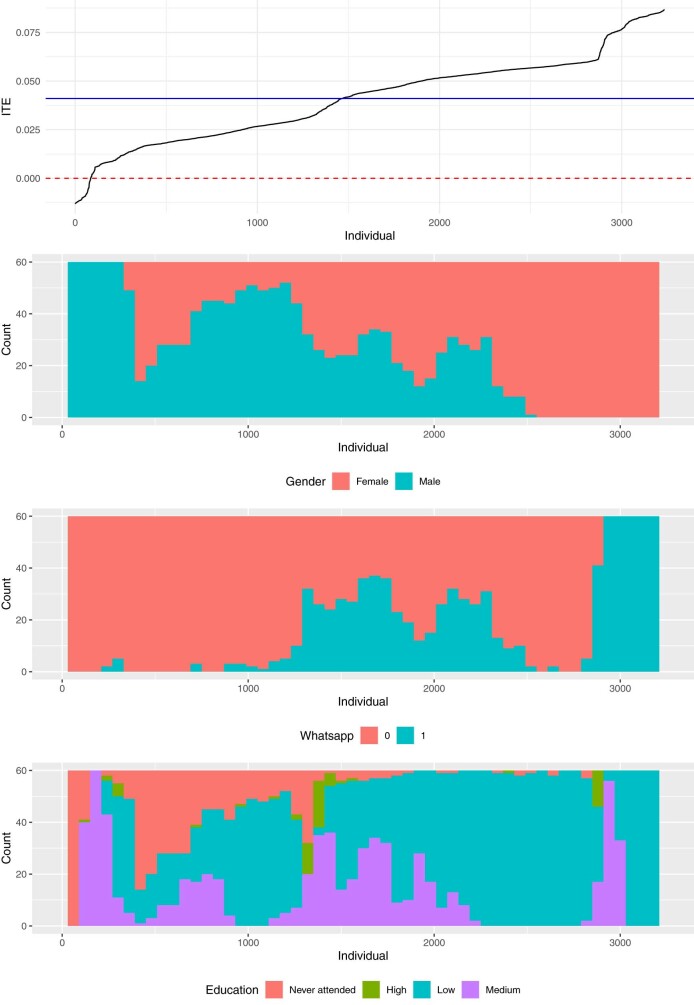
**BART: Heterogeneous Treatment Effects (Gender, WhatsApp and Education)**.

We pre-specified a Bayesian Additive Regression Trees (BART) analysis to test for treatment effect heterogeneity. The BART analyses conducted on reported vaccination outcomes identified three primary candidates for heterogeneity: gender, WhatsApp usage and education. These are presented in Extended Data Figs. 3 and 4. Based on these results, reported in Extended Data Table 6, we estimated interaction terms for these three variables in the vaccine intention models and the reported vaccine models. Women were much more likely to have individual treatment effects (ITEs) above the average for the sample as a whole, whereas male ITEs were more likely to fall below the average. As expected, although imprecisely estimated, the odds ratio for males with respect to the cash treatment arms fell below 1 at 0.87 (CI 0.68, 1.12) for vaccine intention and was 0.82 (CI 0.61, 1.09) for reported vaccination. The BART density plots suggested that participants reporting WhatsApp usage had higher cash treatment effects. Again, the WhatsApp usage estimated odds ratios of 1.02 (CI 0.69, 1.50) for vaccine intention and 1.16 (CI 0.84, 1.60) for reported vaccination in Extended Data Table 6 are directionally consistent although not statistically significant. Finally, the BART density plots for education suggest that the cash treatment effect was lower for those with no formal education, although, while directionally consistent, the effect is not significant for the estimated education interaction terms in Extended Data Table 6.

## Discussion

We conducted a two-stage randomized cluster trial in rural Ghana that assesses the impact of randomly assigned financial incentives on vaccination outcomes. Results from our trial in rural Ghana indicate that financial incentives increased COVID-19 vaccine uptake. The low rate of vaccinations in Africa during the recent COVID-19 pandemic was a serious global public health failure, and our results suggest that financial incentives would have improved vaccination uptake and narrowed the vaccination gap between Africa and the rest of the world.

Non-vaccinated individuals assigned to the financial incentive treatment arms were 9% more likely to express an intention to get the COVID-19 vaccination. This financial incentive-intention result is consistent with other studies that reported financial incentive effects on intention to get vaccinated that varied between 4.2% and 16% (refs. ^8,9,17,18^).

A standard informative health message about the COVID-19 vaccine, or at least the message implemented in this trial, had no effect on vaccination intentions. Others have reported similar null effects, and a recent meta study suggests that online health messaging has not affected willingness to get the COVID-19 vaccine^19^. At least with respect to intentions, a message offering cash incentives for the vaccine will have a much larger marginal effect than a standard health message.

Individuals in the financial incentive treatment arms of our trial were offered cash if they were vaccinated within a 6-week period. Two months after the initial intervention, the average self-reported COVID-19 vaccination rates for respondents in the cash treatment were about 4% higher than those for respondents in the placebo treatment (40% versus 36%). The treatment effect on reported vaccination status is roughly one-half of its effect on vaccination intention. This ratio is consistent with, for example, Dai et al.^20^ who found a 2.5% difference between information treatment effects on booking appointments (6.0%) compared to actual vaccinations (3.5%). Compared to intentions, financial incentives have a weaker, although statistically significant, effect on self-reported vaccination status.

Scaling up such a financial incentive policy requires setting an optimal incentive payment for those getting a COVID-19 vaccine. To give a better sense of the magnitude of the financial incentives used in our study, the high incentive of $10 is equivalent to about 80 Ghana cedis, which is half the reported amount spent on food per week, and 41% of total weekly spending for the average household in our sample. The low incentive of $3 (equivalent to 24 cedis) amounts to about 15% and 12% of the weekly food and total spending, respectively, for the average family in our study.

Our secondary pre-registered hypothesis was that the $10 treatment effect on vaccination uptake would be significantly higher than the $3 incentive. In fact, we found the opposite. Vaccination rates may not respond to higher cash incentives because the $10 payment signals low quality or potential health risks^21,22^. As the Maughan-Brown et al. South African survey highlighted, the potential risks of the vaccine were a major concern of respondents and a substantial barrier to vaccination uptake^23^. The result is also consistent with claims that cash payments that reasonably compensate individuals for the economic costs associated with getting the vaccine are normatively or ethically acceptable^24^. The Maughan-Brown et al. COVID-19 survey in South Africa identified the need to take unpaid time off from work as one of the main barriers to vaccination uptake^23^. Our $3 cash incentive, as opposed to the $10 payment, might have been seen as an acceptable compensation for the costs associated with getting the vaccine. We do, however, need to be cautious in drawing conclusions regarding the impact of varying cash amounts and vaccine uptake because we observed only two price points in this trial.

Clearly context matters. Our results, along with a number of other COVID-19 vaccine experimental trials with financial incentives, suggest considerable variation in the magnitudes of incentives associated with increased vaccine uptake^9,11^. Africa, for example, is certainly a different context than Sweden in this regard. Moreover, as we saw with our Ghana trial, even within a given context, we need to test a range of cash incentives to estimate an informative demand function.

Another concern regarding financial incentive campaigns at scale is the impact that these cash payments may have on individuals who might not be eligible for the incentives or on future vaccination uptake by those who received a financial incentive. Our pre-registered expectation was that proximity to participants who received one of the two cash incentive treatments would depress the vaccine intentions and reported vaccine rates of non-treated individuals. The rural villages in the selected rural districts were quite small; on average, they had a population of 1,268. In each cash-treated village, we sampled 21 individuals, and 75% of them received the cash incentive. This would seem to be a context that would favor spillover effects from the cash treatments. To identify spillover effects from financial incentive campaigns, 25% of the individuals in cash-treated and health-message-treated villages received the placebo treatment. We found no evidence of a negative within-cluster spillover effect. Secondly, we measured the self-reported, and verified, vaccination rates of randomly selected non-treated individuals in cash-treated villages and found no evidence of spillover effects. There is no evidence supporting our pre-registered hypothesis that the cash incentives treatments would depress the vaccination intentions and behaviors of those residing in cash-treated villages who did not receive cash for vaccination. This is consistent with a recent Swedish RCT that found no evidence of a spillover effect of COVID-19 financial incentives on future vaccination uptake^25^.

Although we are confident in our spillover conclusion in the context of our study, we cannot rule out the possibility that, in a larger-scale roll-out of financial incentives, where a substantial proportion, but not all, of a population was offered incentives, there might be negative spillovers on untreated individuals arising from greater diffusion of information.

We explicitly timed the implementation of our incentive trials to correspond with the COVID-19 vaccination campaigns conducted by District Health Offices. COVID-19 vaccinations were available to all villages, and, in most cases, the District Health Offices made the vaccines available in the village communities. This is important because, as a recent trial in rural Sierra Leone demonstrated, by solving the delivery challenges and markedly increasing convenience, COVID-19 vaccine uptake increased by 19% (ref. ^26^). However, we cannot be certain that convenient access to COVID-19 vaccinations was ensured for all trial participants; some may have needed to travel to a nearby health office for the vaccine. Accordingly, we included access as a covariate in the estimations; at least in the case of verified vaccinations, access does not appear to have a statistically significant effect on vaccination uptake. An important future challenge is understanding how the treatment effects of financial incentives, weighted by their overall costs, compare to the relatively costly effects of eliminating delivery logistics at the village level.

Our study has some limitations. First, in each of our sampled villages, we were not able to precisely document the availability of COVID-19 vaccine supplies and the phase of the vaccine roll-out that was occurring when our intervention was implemented. Second, the results could be conditional on the particular design, content and delivery of the video messages. Third, we cannot rule out the possibility that a much larger-scale roll-out of financial incentives might be accompanied by greater diffusion of information and negative spillovers to untreated individuals, although there is no indication from our results that these spillovers are more likely to be negative than positive. Fourth, the context was financial incentives for adults for vaccination against COVID-19 in rural Ghana; treatment effects and spillover effects may be different among different populations (urban settings, for example) and for vaccination against different diseases. Nevertheless, our study is one of the few COVID-19 financial incentive studies that measures actual vaccination outcomes. The results provide insights for the rural Ghana population and may have relevance for the approximately 50% of the overall African population living in non-urban settings^27^.

## Methods

### Trial oversight

The design was approved by the Economics Departmental Research Ethics Committee (DREC) of the Social Science Division of the University of Oxford with the University of Ghana recognizing the ethics oversight of the University of Oxford DREC. The study was registered on the American Economic Association (AEA) registry for randomized social experiments (AEARCTR- 0008775).

The CUREC protocol and relevant amendments are available online (along with the data and code). There was no deviation from the CUREC protocol. Additional details on the design were registered in a statistical analysis plan at 10.1257/rct.8775. The analysis in this paper focuses only on the primary outcomes registered on 10.1257/rct.8775.

The sample, the specific unit of randomization, the randomization methods and the planned analyses were pre-registered on 10 January 2022 before the phase I intervention of the trial. It is a publicly available statistical plan (10.1257/rct.8775). We followed the analysis plan for the primary outcomes outlined in the pre-registered plan.

Here we focus on the direct effects of the intervention on the primary outcomes specified in the AEA registration. The statistical analysis plan also discusses the analysis of pre-registered indirect spillover effects that were incorporated into the initial design. A more extensive follow-up campaign aimed at measuring additional spillover effects will be conducted along with various supplementary analyses. Results of these are left for follow-on work.

### Intervention

The experiment has four treatments that are delivered in a short video:

- Treatment 1: a 45-s placebo video that provides general information about the benefit of using solar power to charge household electrical appliances.
- Treatment 2: a 45-s standard health COVID-19 vaccine promotional and information video (modeled on the videos produced by the US Centers for Disease Control and Prevention (CDC)).
- Treatment 3: low cash incentive treatment. The first 30 s are identical to the health video; the last 15 s inform viewers that they will earn $3 (20 Ghana cedis) if they receive the COVID-19 vaccine within the next 6 weeks.
- Treatment 4: high cash incentive treatment. The first 30 s are identical to the health video; the last 15 s inform viewers that they will earn $10 (60 Ghana cedis) if they receive the COVID-19 vaccine within the next 6 weeks.

The four treatment videos are available at https://www.youtube.com/watch?v=PeM1cpCU0bA&list=PLBGbIwQfB9sez5Ww6xcKmwBQa45Z3Vvwd. Individuals viewed the videos on a tablet that was presented to them by an enumerator. The randomizaton of video presentations was managed by a downloaded Qualtrics script from the Candour server. The interventions were conducted in person by enumerators from the University of Ghana.

Individuals were compensated with 5 Ghana cedis immediately after this phase I intervention; they received an additional 5 Ghana cedis for their participation in the phase II/III post-treatment survey. Signed consent to participate in the RCT was obtained from each participant; the full text of the signed consent can be found in the text of the phase I questionnaire.

### Trial design, eligibility, randomization and recruitment

Working with the district offices of the Ministry of Health, we selected one region (Central) and six districts that were scheduled to have COVID-19 vaccine supplies made available in January–February 2022. Vaccine supplies were being made available on a district-by-district basis in Ghana. The six districts in our sample are:

- District 1: Gomoa West – Central Region
- District 2: Asikuma Odoben Brakwa – Central Region
- District 3: Twifo Ati-Morkwaa – Central Region
- District 4: Assin North – Central Region
- District 5: Asugyaman – Central Region
- District 6: Upper Manya Krobo – Central Region

To ensure that we would have adequate power for the COVID-19 vaccine outcomes, we randomized at the village level to generate experimental variation for each set of outcomes. The CONSORT diagram (Fig. 1) describes the design and the allocation of clusters to each arm.

Working with our partners in each district, we identified the list of district villages that could feasibly be enumerated by our enumeration team (the primary consideration here was either road access or the quality of the road access). Then, within each district, we generated a complete list of the villages that are candidates for enumeration. The villages were then ranked according to their population size (population statistics provided by the 2010 Ghana census). We then formed groups of four villages by putting four consecutive villages on these lists in the same quadruplet. In a typical district, we would have approximately 50 quadruplets.

In each district, we randomly selected 13 quadruplets with probabilities weighted by the quadruplet’s share of the total population of the villages being considered in the district. This initial sample of quadruplets was then adjusted in consultation with the district health officials. The adjusted selection criteria are driven, in part, by cost considerations; for budget reasons, we were constrained to ensure reasonable travel distances between the four village clusters. We also needed to ensure, in collaboration with district health officials, that the COVID-19 vaccines were readily available in the four village clusters selected. Within each of the chosen quadruplets, we randomly selected one village for the placebo treatment; one for the standard health video; one for the low cash incentive video; and one for the high cash incentive video. This resulted in a total sample of 310 villages from the six different districts. Within each of the three treatment villages (health, low cash and high cash), 75% of the participants were randomly assigned to the village treatment, and 25% of the participants were randomly assigned to a placebo video. The complete list of villages and treatment assignments is available in the Supplementary Information.

Enumerator IDs were assigned to village clusters. At the beginning of each enumeration day, enumerators downloaded their Qualtrics questionnaire assignments with the appropriate embedded videos. At the end of the day, these completed questionnaires were uploaded to the Candour server. No survey data were left resident on the individual tablets. Enumerators were provided with a random walk protocol for selecting households in each village; the detailed protocol was included in the pre-registration and is available at 10.7910/DVN/X5MBHP.

Within each of the 310 villages, we randomly sampled 21 individuals. Households were randomly selected, and then, within households, we randomly selected individuals 18 years of age and older with the condition that they had not received the COVID-19 vaccine. There are four treatment arms with 80 clusters in each treatment arm. We sampled 5,900 in the first phase. The power calculations in our pre-registration assumed 300 villages in the design and at least 25 respondents per village. In this case, we are powered to detect an effect size of 0.06. All of the simulations—villages ranging in number from 200 to 400 with as few as 20 treated participants per village—were powered at 0.80 to detect an effect size of 0.12. We anticipated that our design, with four treatment arms, 312 villages and 21 treated participants per village, would be powered at approximately 0.80 to detect an effect size as small as 0.06.

As part of phase III of the trial, we implemented a recruitment strategy for non-treated participants. For these non-treated participants, we collected information on their COVID-19 vaccination status as well as their demographics, their social network density and how and whether they learned about financial incentives or about getting the vaccination. We interviewed non-treated participants in all villages that were visited as part of phase III. The group of non-treated households within treated villages were selected by a similar random walk that was implemented in the initial treatment phase I.

### Outcomes

Three outcome measures were collected in this RCT: intention to get vaccinated, reported vaccination and verified vaccination status.

### Vaccination intentions

In phase I of the trial, immediately after the video treatment, individuals were asked for their intention to get vaccinated in the next 6 weeks. This serves as our measure of Vaccine Intention.

### Reported vaccine status

In phase II of the trial, approximately one-half of the individuals were contacted by telephone and reported their vaccination status. This serves as our measure of Reported Vaccination. In phase III, the other half of the individuals were contacted in person to obtain Reported Vaccination.

### Validated vaccine status

In phase IV, the staff of the six District Health Offices were supplied with a list of the 5,900 phase I treated participants and of the approximately 1,101 participants who made up the spillover sample from phase III. This list included names, village, address if available and telephone numbers. Health officials were blinded to the treatment status of the individuals on the lists that we provided for each District Health Office. The District Health Offices returned a list indicating: (1) whether each of the trial participants had received at least one dose of a COVID-19 vaccine; (2) when they received their first dose; and (3) if the information was not available in the District Health Office. In addition to indicating ‘yes’ or ‘no’ and date of first vaccination, District Health Office staff also indicated ‘no information’ or ‘N/A’. We only include in the final sample, used for the analysis, individuals who received either a ‘yes’ or a ‘no’. Note that the two cash treatments specified that individuals would receive a cash payment if they received a vaccination within the next 6 weeks. Accordingly, for the analysis reported in the text, trial participants who were vaccinated after April 2022, which is approximately 2 months after the video intervention, are coded as ‘no’. These verified vaccination data were obtained in phase IV during the period 15 October to 30 November 2022.

### Covariates

Individual-level covariate measures were obtained from questionnaires administered in each of the three phases of the trial (the different versions of the surveys are available in the Supplementary Information). All individuals were asked questions regarding their vaccination status and vaccination intentions. The covariate controls in the estimation are based on a battery of demographic questions (summarized in Table 1) that were asked in all phases of the trial. For the estimation of spillover effects, we included in the phase II and phase III survey measures of network density that draw on a growing literature on contagion effects^28^. In the phase III questionnaires administered to non-treated individuals, we included a battery of questions measuring COVID-19 vaccine information acquisition. Details on question wording are available in the Supplementary Information.

### Access

The distance between individuals and the nearest district health clinic is our measure of access to a COVID-19 vaccine. This is first based on the geo-location of individuals. In most cases, we could identify the individual’s geo-location based on the geo-location of the tablet when phase I treatment intervention was conducted with the treated individuals and when phase III was conducted with the non-treated individuals. Secondly, we collected the geo-location of all clinics in the six Ghana districts that were sampled in this trial. Using the R function distHaversine, we calculated the shortest distance between each participant and each of these district health clinics^29^. The access variable in the results measures the distance between each participant and the district health clinic that is closest to the participant.

### Spillover treated

In the villages assigned to the three treatment arms—health message, low cash and high cash—we randomly assigned 25% of the individuals to a placebo video treatment. These ‘embedded placebo’ participants are identified by the following dummy variables: HealthPlacebo (takes a value of 1 for participants viewing the placebo video in a village assigned to the health treatment); LowCashPlacebo (takes a value of 1 for participants viewing the placebo video in a village assigned to the low cash treatment); HighCashPlacebo (takes a value of 1 for participants viewing the placebo video in a village assigned to the high cash treatment); and CashPlacebo (takes a value of 1 for participants viewing the placebo video in a village assigned to either the low or high cash treatment). These dummy variables for the ‘embedded’ placebo individuals will measure spillover effects.

### Spillover untreated

There is a sample of untreated individuals that we will include in the model estimation to estimate possible spillover effects from the vaccine cash incentive treatments. The binary UntreatedTreated variable identifies individuals who were in treated villages but received no treatment and were surveyed after treatment.

### Statistical analysis

The analysis was performed by original assigned treatment group (intention to treat), following the statistical plan.

### Effect on verified vaccination status

Our primary pre-registered conjecture is that cash incentives would have a positive effect on vaccine update. We estimate the following logistic regression and hypothesize that *β*_2_ > 0 and *β*_2_ > *β*_1_:

1 $${{\rm{Vaccinated}}}_{ic}={\beta }_{0}+{\beta }_{1}{{\rm{Health}}}_{ic}+{\beta }_{2}{{\rm{Cash}}}_{ic}+\omega {{\bf{X}}}_{ic}+{{\epsilon }}_{i}$$

where:

- Vaccinated_*ic*_ has a value of 1 if individual *i* in cluster *c* is vaccinated within the 6-week period after the video intervention.
- Health_*ic*_ has a value of 1 if individual *i* in cluster *c* is treated with a standard CDC health message about COVID-19 vaccinations.
- Low cash_*ic*_ has a value of 1 if individual *i* in cluster *c* is treated with the video offering a low cash incentive ($3).
- High cash_*ic*_ has a value of 1 if individual *i* in cluster *c* is treated with the video offering a high cash incentive ($10).
- Cash_*ic*_ has a value of 1 if individual *i* in cluster *c* is treated with the video offering a cash incentive (either $3 or $10).
- **X**_*ic*_ are covariate controls: age, gender and education.

Standard errors are adjusted for clustering at the village level. CIs are constructed using a *t*-distribution.

A secondary conjecture is that the higher cash incentive would have a larger effect than the lower cash incentive on vaccine uptake. We estimate the following logistic regression and hypothesize that *β*_3_ > *β*_2_:

2 $$\begin{array}{l}{{\rm{Vaccinated}}}_{ic}={\beta }_{0}+{\beta }_{1}{{\rm{Health}}}_{ic}+{\beta }_{2}{\rm{Low}}\,{{\rm{Cash}}}_{ic}\\+{\beta }_{3}{\rm{High}}\,{\rm{Cash}}_{ic}+\omega {{\bf{X}}}_{ic}+{{\epsilon }}_{ic}\end{array}$$

### Effect on vaccine intention/reported vaccination

Our secondary pre-registered conjecture is that cash incentives would have a positive effect on both Vaccine Intention and Reported Vaccination:

3 $${\rm{Vaccine}}\,{{\rm{Intention}}}_{ic}={\beta }_{0}+{\beta }_{1}{{\rm{Health}}}_{ic}+{\beta }_{2}{{\rm{Cash}}}_{ic}+\omega {{\bf{X}}}_{ic}+{{\epsilon }}_{ic}$$

4 $${\rm{ReportedVaccination}}_{ic}={\beta }_{0}+{\beta }_{1}{{\rm{Health}}}_{ic}+{\beta }_{2}{{\rm{Cash}}}_{ic}+\omega {{\bf{X}}}_{ic}+{{\epsilon }}_{ic}$$

### Spillover treated effect

The following regression estimates spillover effects by including the two ‘embedded placebo’ dummy variables: HealthPlacebo and CashPlacebo. In our pre-registration, we hypothesized that vaccination rates of individuals receiving the placebo treatment in villages assigned to a cash treatment would be depressed. In this specification, we are testing whether the 25% of placebo individuals in villages assigned the health message or cash treatments have vaccination rates that are different than those for individuals in the villages assigned to the placebo treatment. Our expectation is that the overall average vaccination rates will be depressed for the 25% in the cash-treated villages, hence the expectation that *β*_4_ < 0.

5 $$\begin{array}{l}{{\rm{Vaccinated}}}_{ic}={\beta }_{0}+{\beta }_{1}{{\rm{Health}}}_{ic}+{\beta }_{2}{{\rm{HealthPlacebo}}}_{ic}+{\beta }_{3}{{\rm{Cash}}}_{ic}\\+{\beta }_{4}{{\rm{CashPlacebo}}}_{ic}+\omega {{\bf{X}}}_{ic}+{{\epsilon }}_{ic}\end{array}$$

We also restrict the regression analysis to the placebo participants and estimate the following equation:

6 $${{\rm{Vaccinated}}}_{ic}={\beta }_{0}+{\beta }_{1}{{\rm{HealthPlacebo}}}_{ic}+{\beta }_{2}{{\rm{CashPlacebo}}}_{ic}+\omega {{\bf{X}}}_{ic}+{{\epsilon }}_{ic}$$

Again, our pre-registered hypothesis was that *β*_2_ < 0.

### Spillover untreated effect

For the sample of untreated individuals in treated village clusters, we estimate the following equation to test our spillover conjectures:

7 $${{\rm{Vaccinated}}}_{ic}={\beta }_{0}+{\beta }_{1}{{\rm{Health}}}_{ic}+{\beta }_{2}{{\rm{Cash}}}_{ic}+\omega {{\bf{X}}}_{ic}+{{\epsilon }}_{ic}$$

Again, our pre-registered hypothesis was that *β*_2_ < 0. Those untreated individuals most proximate to the cash incentive treatment would have relatively depressed vaccination rates.

### Balance

Balance on covariates is assessed by comparing their standardized mean differences (raw differences in proportion for binary variables) across the four treatment arms. We also compare these unadjusted differences with those obtained when the sample is weighted using propensity score matching. These are generated for the sample of 5,900 participants interviewed in phase I. The detailed results and comparisons are generated by the R program Cobalt^30^. Extended Data Fig. 1 compares, for the full phase I sample, for each treatment pair, the standardized mean differences for the unadjusted and adjusted covariates. We employ indicative balance tolerance levels of 0.1 in Extended Data Fig. 1 (the vertical dotted lines)^31–33^. With the exception of only a couple of the 18 covariates across the six comparisons, the standardized mean differences for the unadjusted sample fall within this 0.1 threshold.

### Attrition

The phase I design of the study measures a vaccine intention treatment effect immediately after participants view one of the four video treatment arms. None of the individuals terminated the survey after viewing the randomly assigned video treatment. Therefore, potentially confounding incomplete survey responses is not a concern here. We do, however, observe attrition between treatment at baseline (phase I) and post-treatment (phase II and phase III) and between phase I baseline and verified vaccination status (phase IV). Extended Data Table 2 presents baseline phase I descriptive statistics for those who were contacted in phase II/III, and similar descriptives are for those who were not contacted in phase II/III. A profile of attrition is presented in Extended Data Table 3. In the follow-up phase II/III, we contacted 4,101 of the original 5,900 individuals; overall attrition is about 30%, and this is reasonably similar across treatment arms.

Extended Data Table 3 compares the baseline phase I intended vaccination rates of participants, in each of the four treatment arms, who were, and were not, contacted in the post-treatment phase II/III. In the low cash treatment, those not contacted in post-treatment had higher baseline vaccine intention rates (86.5%) than those contacted (79.6%). In the high cash arm, those non-contacted had slightly lower baseline vaccine intentions (76.7%) than those contacted (78.9%). The non-contacted in the health treatment arm had a baseline vaccine intention rate of 73.3% compared to 72.3% for those who were contacted in post-treatment. For the placebo arm, those non-contacted in post-treatment have lower vaccine intention rates (69.4%) than those who were contacted in post-treatment (71.0%).

Extended Data Table 3 compares the average vaccine intention treatment effects of the three interventions compared to placebo for those who were, and were not, contacted in post-treatment. The non-contacted in the health arm had higher average treatment effects than those who were contacted (3.9 versus 0.48); for low cash, it was 17.1 versus 7.9; and for high cash, it was 7.3 versus 7.1. Similar patterns are reported in Extended Data Table 3, which presents similar analyses for ‘compliers’ versus ‘non-compliers’ in phase IV.

We employ multiple empirical strategies to test for attrition bias in our estimated treatment effects. First, we generate differential attrition rate tests to assess whether the rates of attrition are statistically significantly different across treatment and control groups. Extended Data Table 4 presents the attrition test results. Model 1 is a logit regression of compliance in the follow-up phase II/III (individuals have a value of 1 if they complied and 0 if they did not) on treatment arm. None of the treatment arms have statistically significant coefficients. Model 2 is a similar logit regression that also includes covariates. In this case, the health message arm has a significant coefficient. Model 3 includes fixed effects for village clusters and has results very similar to model 2. On balance, there is no compelling evidence here of bias resulting from attrition. Extended Data Table 4 replicates this analysis, comparing the phase I and phase IV (Vaccination Verification) samples. The negative coefficients for the three treatment arm dummy variables (health, low cash and high cash) are not statistically different from each other. Thus, the primary imbalance here concerns the placebo treatment arm.

We use the complete set of demographic measures collected in the pre-treatment survey to model attrition. We then re-estimate treatment effects employing inverse probability weighting and report the results in Supplementary Table 1. The results are similar to those that we reported in Table 3 in the main text. Estimated odds ratios for the cash incentive treatment are smaller, although statistically significant, for all three outcome variables.

In phase IV of the trial, we enlisted district health officials to verify the vaccination status of the trial participants. We were able to verify the vaccination status of 3,075 from the original 5,900 phase I intervention. The success of these vaccination verification efforts varied across the six health districts. Accordingly, we assess the sensitivity of our estimations to the different district verification samples. Supplementary Table 4 reports the results of dropping each district from the multi-variable model of verified vaccination status. For the most part, the low cash treatment effect is robust to dropping individual district samples. The exception is that when we drop District 4, the odds ratio is 1.5 compared to 1.75 in the full model, and, because we lose about 600 observations, the confidence bands are wide.

Analyses were performed using R version 4.2.0, including the following packages (versions): htmlTable, stargazer, data.table, DescTools, ggpubr, stats (4.0.3), tidyverse (1.3.0), estimatr (0.28.0), readr (1.4.0), dplyr (1.0.5), lubridate (1.7.10), hdm (0.3.1), car (3.0.10), MASS (7.3.53), sandwich (3.0.0), misty (0.4.6), foreign (0.8.80), readxl (1.3.1), mlogit (1.1-1), nnet (7.3), aod (1.3.2), RVAide- Memoire (0.981-2) and quantreg (5.75) cobat (4.4.1), miceadds (3.16-18) and BART (2.9.4). Data collection was conducted using Qualtrics First Release 2005 (https://www.qualtrics.com/).

### Inclusion and ethics statement

The initial concept of a Ghana COVID-19 Vaccine Incentive research project was the product of conversations between Professor Asiedu (University of Ghana) and Raymond Duch (University of Oxford). Asiedu and Duch have been working together for many years on field experiment projects in Ghana. The actual study design—including, most importantly, selection of target population, sampling strategy, video treatments, financial incentives and vaccination outcome measures—was developed in close collaboration with Professor Asiedu and his PhD student, Dorcas Sowah. All of the data and intellectual property associated with the project are shared with Professor Asiedu and the University of Ghana. Both Professor Asiedu and Ms. Sowah are co-authors on resulting publications.

Our local partners in the project are the Health District Offices in the six regional districts where the research was conducted. We were able to implement the Ghana COVID-19 Vaccine Incentive research project because our local partners considered it extremely relevant to the challenges faced in vaccinating their rural populations. More broadly, though, the goal of assessing whether financial incentives could significantly increase vaccine uptake was very much relevant for health policymakers in Ghana and Africa overall because of the relatively slow pace of vaccine uptake in the region. Several leading policymakers advocated exploring the role that financial incentives could play, and there was very little evidence from LMICs regarding the impact that such a policy might have.

To ensure that the project design and roll-out incorporated maximum input from local partners, Raymond Duch met regularly in person with the district health officials to report the ongoing results and to determine what changes in the design and implementation were necessary to ensure maximum local relevance of the study results. The local University of Ghana team and the other regional research teams met weekly to ensure, again, that our design and implementation were locally relevant.

The roles and responsibilities among collaborators were agreed upon ahead of the research project. These were outlined in documents jointly prepared by the University of Ghana and University of Oxford teams. They included roles and responsibilities for the Ghana academic team, the Oxford academic team, the other regional academic teams and the local Ghana health district teams. Capacity building was an important feature of the research implementation strategy. The project included support for capacity building at the individual Ghana health district level; resources and funding for building the research infrastructure of the University of Ghana team (which includes a large team of student enumerators and researchers); and career development opportunities for the senior members of the University of Ghana research team.

The research would not have been severely restricted or prohibited in the Ghana setting where the research took place. As we pointed out in our ethics submission to the University of Oxford, the treatments consisted only of encouragements for the treated individuals to get a COVID-19 vaccine. All participants in the study were compensated for their participation.

Because of time constraints, given the urgency of having our study go into the field as the COVID-19 vaccines were being distributed in our targeted rural districts, we opted for only a single high-intensity ethics review at the University of Oxford (CUREC 2). This was a joint decision of the local research team and the Oxford research team. Professor Asiedu from the University of Ghana was one of the co-principal investigators included in the University of Oxford ethics submission.

The research resulted in no stigmatization, incrimination, discrimination or other personal risks to participants. In addition, we adopted very strict measures to ensure that the information we collected was anonymized and that we followed strict University of Oxford procedures for data protection.

The research was implemented during the COVID-19 pandemic, and we reviewed with local partners and Ghana district health officials the appropriate measures that needed to be put into place to protect both the enumerators who were conducting the interventions and the study participants. We have a detailed discussion of these measures in our ethics submission to the University of Oxford. In addition, we provided detailed instructions to enumerators regarding COVID-19 symptoms and COVID-19 protection measures; these were provided in initial documents on the tablet surveys that were administered. Raymond Duch also briefed enumerators in person on the necessary COVID-19 measures that needed to be adopted. Participants were also provided with COVID-19 instructions; for example, they were questioned about COVID-19 symptoms and instructed regarding masking and social distancing.

In preparing this manuscript, the authors were very careful to ensure that all local and regional research relevant to the study and its results were taken into account in citations.

### Reporting summary

Further information on research design is available in the Nature Portfolio Reporting Summary linked to this article.

## Online content

Any methods, additional references, Nature Portfolio reporting summaries, source data, extended data, supplementary information, acknowledgements, peer review information; details of author contributions and competing interests; and statements of data and code availability are available at 10.1038/s41591-023-02670-4.

### Supplementary information

Supplementary Information Table of contents, Supplementary Tables 1–5, three versions of the questionnaire and list of village clusters.

Reporting Summary

## Figures and Tables

**Extended Data Table 1:**
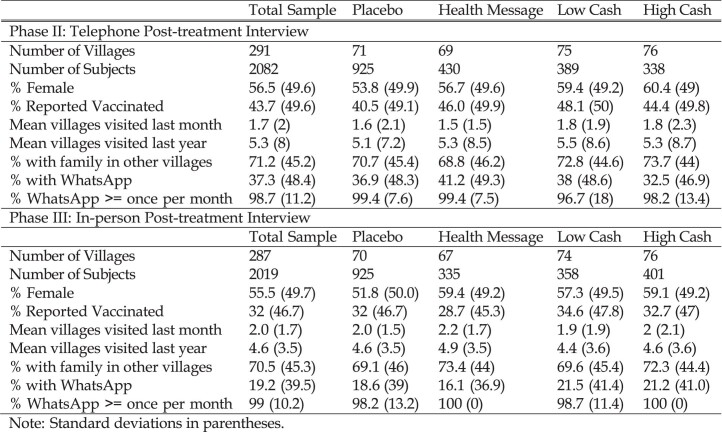
Descriptives phase II and III (post-treatment telephone and in person: reported vaccinations)

**Extended Data Table 2:**
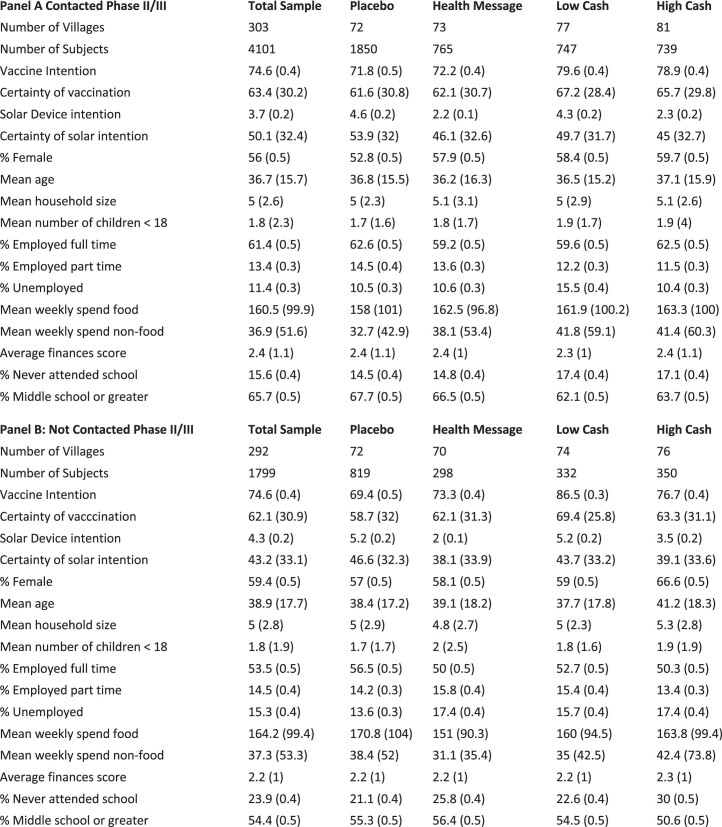
Baseline (phase I) descriptive statistics for respondents contacted/not contacted in phase II/III post-intervention follow-up

**Extended Data Table 3:**
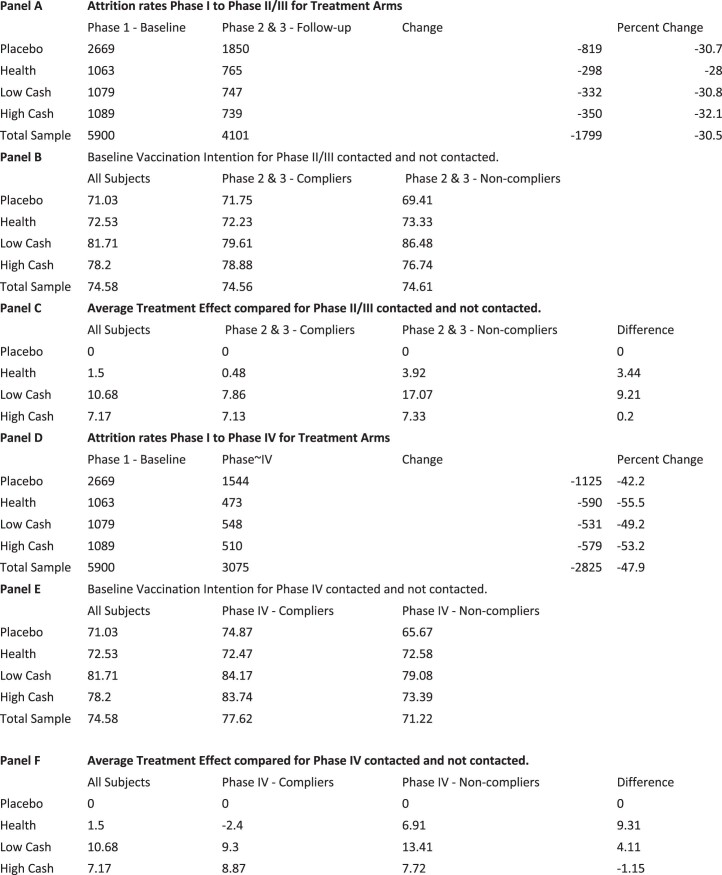
Attrition rates and analyses for treatment arms, phase I to phase IV

**Extended Data Table 4:**
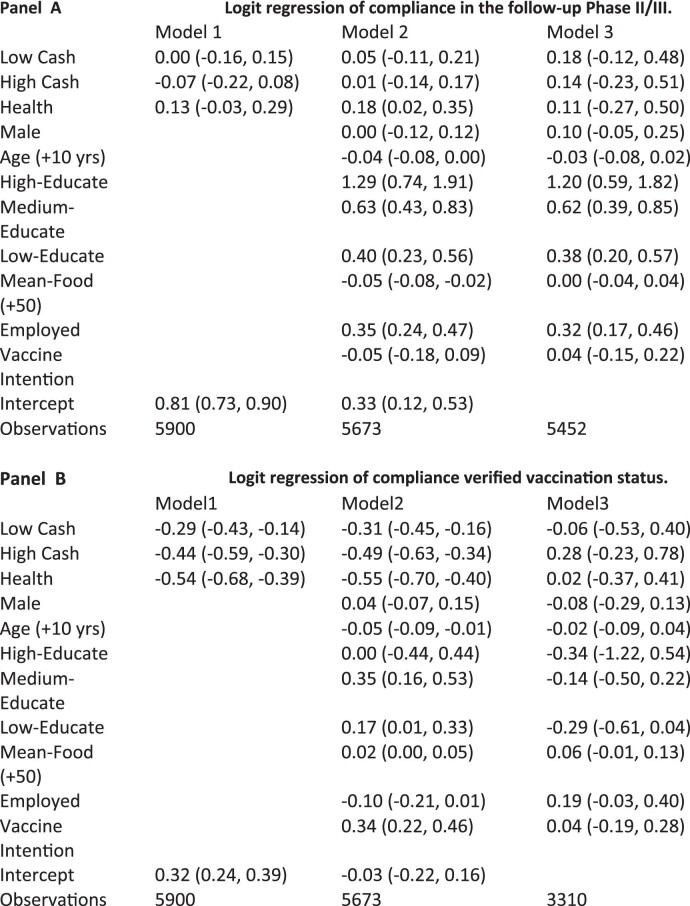
Attrition rate tests

**Extended Data Table 5:**
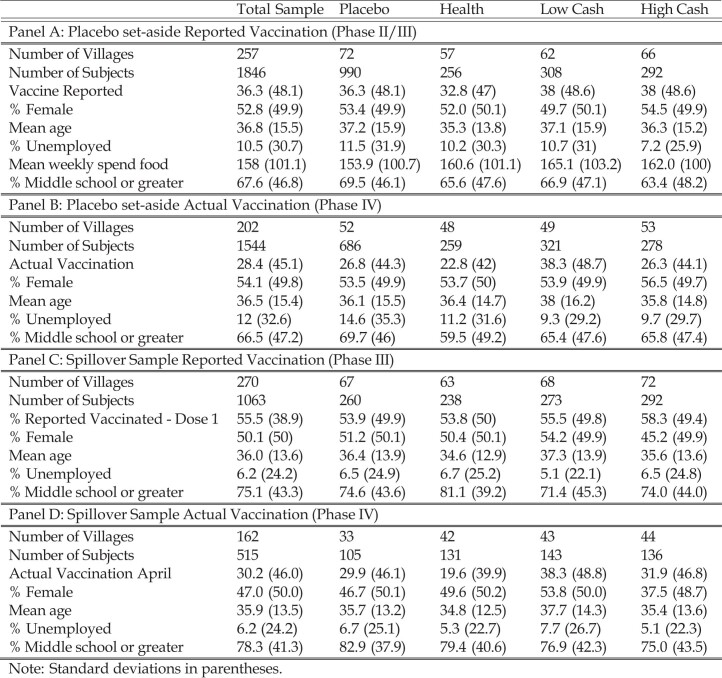
Sample descriptives: placebo set-aside and spillover samples

**Extended Data Table 6:**
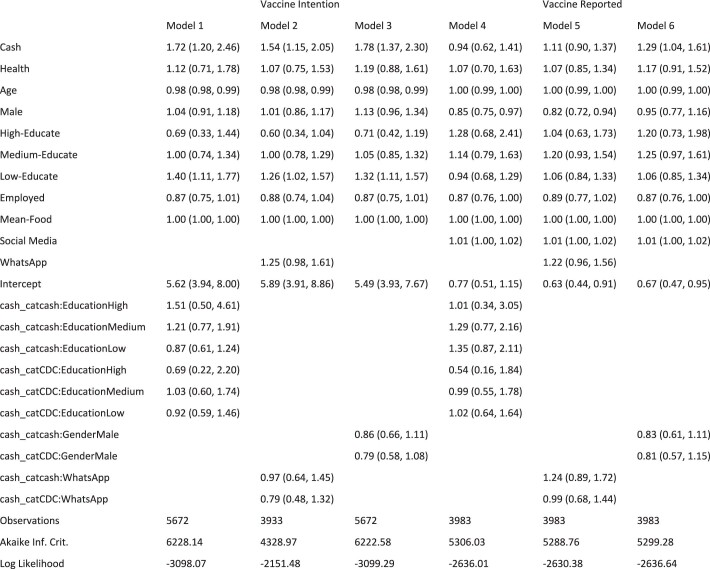
Heterogeneity interaction models

## Data Availability

All anonymized data used in the analysis are publicly available at 10.7910/DVN/X5MBHP. All data are shared in a public registry (10.7910/DVN/X5MBHP). The data are freely accessible at 10.7910/DVN/X5MBHP.

## Code availability

All codes are available at 10.7910/DVN/X5MBHP. The code is freely accessible at 10.7910/DVN/X5MBHP.
