# Author Correction: Financial incentives for COVID-19 vaccines in a rural low-resource setting: a cluster-randomized trial

**DOI:** 10.1038/s41591-023-02772-z

**Published:** 2023-12-22

**Authors:** Raymond Duch, Edward Asiedu, Ryota Nakamura, Thomas Rouyard, Alberto Mayol, Adrian Barnett, Laurence Roope, Mara Violato, Dorcas Sowah, Piotr Kotlarz, Philip Clarke

**Affiliations:** 1 Nuffield College, University of Oxford, Oxford, UK; 2 Edward Asiedu and Dorcas Sowah University of Ghana Business School, University of Ghana, Accra, Ghana; 3 Hitotsubashi Institute for Advanced Study, Hitotsubashi University, Tokyo, Japan; 4 City University of New York (CUNY) Graduate School of Public Health & Health Policy, New York, NY USA; 5 Department of Public Administration, FAE University of Santiago Chile, Santiago, Chile; 6 Queensland University of Technology, Brisbane, Queensland Australia; 7 University of Oxford, Oxford, UK; 8 University of Melbourne, Melbourne, Victoria Australia

**Keywords:** Social sciences, Infectious diseases

Correction to: *Nature Medicine* 10.1038/s41591-023-02670-4, published online 27 November 2023.

In the version of the article initially published, there was an error in the “Trial design, eligibility, randomization and recruitment” section: the sentence now reading “…the detailed protocol was included in the pre-registration and is available at 10.7910/DVN/X5MBHP” had the wrong link. This has now been corrected in the HTML and PDF versions of the article.

