# Supplementary material for: Financial incentives for COVID-19 vaccines in a rural low-resource setting: a cluster-randomized trial
Source: Nat Med. 2023 Nov 27;29(12):3193–202. doi: 10.1038/s41591-023-02670-4 (PMC10719106; doi:10.1038/s41591-023-02670-4)
Supplement: Supplementary file 1 — Table of contents, Supplementary Tables 1–5, three versions of the questionnaire and list of village clusters. [file 41591_2023_2670_MOESM1_ESM.pdf]

nature medicine

Article https://doi.org/10.1038/s41591-023-02670-4

Financial incentives for COVID-19 vaccines in a rural low-resource setting: a cluster-randomized trial

In the format provided by the authors and unedited

Table of Contents

Supplementary Table S1: ***2***

Estimated Treatment Effects with Inverse Probability Weights ....

Supplementary Table S2: ***3***

Spillover odds ratios for sample restricted to placebo treated participants ....

Supplementary Table S3: ***4***

P-values compared to Randomization Inference ....

Supplementary Table S4: ***5***

Verified Vaccination Regression: Robust to District Samples ....

Supplementary Table S5: ***6***

Spillover Regression Estimates Including Population Size Interactions. ....

Ghana Vaccine Incentives Phase I Survey: ***7-34***

Phase II: Ghana Incentives Post Treatment Survey: ***35-48***

Phase III: In-person Post-treatment Survey: ***48-62***

Phase III: Ghana Financial Incentives Spillover Survey: ***63-88***

Sampled Village Clusters: ***89-97***

Supplementary Table S1: Estimated Treatment Effects with Inverse Probability Weights

	Intention	Intention IPW	Reported	Reported IPW	Actual	Actual IPW
Cash	1.79 (1.45, 2.22)	1.09 (1.06, 1.12)	1.21 (1.02, 1.45)	1.05 (1.01, 1.08)	1.47 (0.92, 2.36)	1.07 (1.02, 1.11)
Health	1.15 (0.87, 1.51)	1.01 (0.97, 1.05)	1.09 (0.89, 1.34)	1.02 (0.98, 1.06)	0.70 (0.34, 1.42)	0.95 (0.90, 1.00)
Access (+5)	1.04 (0.89, 1.21)	1.01 (0.99, 1.02)	0.95 (0.86, 1.06)	0.99 (0.97, 1.01)	1.18 (0.78, 1.80)	1.03 (1.00, 1.06)
Age (+10 yrs)	0.83 (0.79, 0.87)	0.96 (0.95, 0.97)	0.96 (0.92, 1.01)	0.99 (0.98, 1.00)	1.03 (0.95, 1.13)	1.01 (0.99, 1.02)
Male	1.10 (0.97, 1.26)	1.02 (0.99, 1.04)	0.87 (0.76, 1.00)	0.97 (0.94, 1.00)	0.97 (0.75, 1.27)	1.00 (0.96, 1.04)
High-Educate	0.68 (0.40, 1.17)	0.90 (0.81, 1.00)	1.15 (0.69, 1.91)	1.03 (0.92, 1.16)	1.85 (0.77, 4.46)	1.09 (0.95, 1.26)
Medium-Educate	0.99 (0.79, 1.23)	0.99 (0.94, 1.03)	1.15 (0.90, 1.48)	1.03 (0.98, 1.09)	1.78 (1.18, 2.71)	1.10 (1.03, 1.17)
Low-Educate	1.26 (1.06, 1.51)	1.03 (0.99, 1.08)	1.01 (0.80, 1.27)	1.00 (0.95, 1.05)	1.27 (0.87, 1.86)	1.04 (0.98, 1.10)
Employed	0.91 (0.79, 1.06)	0.99 (0.97, 1.02)	0.91 (0.79, 1.05)	0.98 (0.95, 1.01)	1.14 (0.89, 1.47)	1.02 (0.99, 1.06)
Mean-Food (+50)	0.94 (0.90, 0.98)	0.99 (0.98, 1.00)	1.00 (0.97, 1.04)	1.00 (0.99, 1.01)	1.03 (0.95, 1.13)	1.01 (1.00, 1.01)
Social Media (+10)			1.09 (0.98, 1.22)	1.02 (1.00, 1.05)	1.06 (0.91, 1.23)	1.01 (0.98, 1.04)
District 2	1.62 (1.10, 2.39)	1.06 (1.01, 1.10)	1.31 (1.02, 1.68)	1.07 (1.02, 1.13)	0.03 (0.01, 0.10)	0.51 (0.45, 0.58)
District 3	1.30 (0.79, 2.13)	1.05 (1.00, 1.11)	1.05 (0.79, 1.41)	1.01 (0.95, 1.07)	0.04 (0.02, 0.11)	0.53 (0.47, 0.61)
District 4	0.76 (0.53, 1.09)	0.93 (0.89, 0.97)	1.07 (0.82, 1.40)	1.02 (0.96, 1.07)	0.11 (0.04, 0.31)	0.65 (0.57, 0.74)
District 5	0.69 (0.48, 1.01)	0.93 (0.89, 0.97)	0.66 (0.52, 0.83)	0.91 (0.86, 0.95)	0.01 (0.00, 0.02)	0.43 (0.38, 0.50)
District 6	0.44 (0.30, 0.65)	0.82 (0.78, 0.87)	0.50 (0.36, 0.69)	0.86 (0.81, 0.91)	0.15 (0.05, 0.41)	0.70 (0.60, 0.81)
Observations	5644	3912	3957	3957	2146	2146

Supplementary Table S2: Spillover odds ratios for sample restricted to placebo treated participants.

	Vaccine Intention	Vaccine Reported	Actual Vaccination
CashPlacebo	1.21 (0.86, 1.72)	1.03 (0.77, 1.38)	1.03 (0.77, 1.38)
HealthPlacebo	0.90 (0.52, 1.56)	0.81 (0.54, 1.22)	0.81 (0.54, 1.22)
Access (+5)	1.01 (0.85, 1.21)	0.90 (0.78, 1.04)	0.90 (0.78, 1.04)
Age (+10 yrs)	0.80 (0.73, 0.87)	0.98 (0.91, 1.05)	0.98 (0.91, 1.05)
Male	1.26 (1.00, 1.59)	1.00 (0.81, 1.24)	1.00 (0.81, 1.24)
High-Educate	0.54 (0.22, 1.32)	1.38 (0.71, 2.66)	1.38 (0.71, 2.66)
Medium-Educate	0.92 (0.64, 1.33)	1.09 (0.74, 1.59)	1.09 (0.74, 1.59)
Low-Educate	1.26 (0.91, 1.74)	0.96 (0.67, 1.38)	0.96 (0.67, 1.38)
Employed	1.02 (0.79, 1.32)	0.87 (0.71, 1.07)	0.87 (0.71, 1.07)
Mean-Food (+50)	0.93 (0.86, 0.99)	0.99 (0.94, 1.04)	0.99 (0.94, 1.04)
Social Media (+10)	1.00 (0.83, 1.21)	1.14 (0.97, 1.35)	1.14 (0.97, 1.35)
District 2	1.08 (0.51, 2.26)	1.45 (0.94, 2.22)	1.45 (0.94, 2.22)
District 3	0.82 (0.35, 1.89)	1.16 (0.77, 1.73)	1.16 (0.77, 1.73)
District 4	0.43 (0.21, 0.88)	1.23 (0.76, 1.98)	1.23 (0.76, 1.98)
District 5	0.38 (0.19, 0.77)	0.67 (0.45, 1.01)	0.67 (0.45, 1.01)
District 6	0.22 (0.10, 0.46)	0.60 (0.38, 0.93)	0.60 (0.38, 0.93)
Intercept	4.98 (2.48, 10.01)	0.66 (0.44, 0.99)	0.66 (0.44, 0.99)
Observations	1709	1785	1081
Akaike Inf. Crit.	1895	2317	1163
Log Likelihood	-930	-1141	-565

Supplementary Table S3: P-values Compared to Randomization Inference.

Outcome	Treatment	Observed P-value	RI P-value
Intention	Health	0.6973	0.6659
Intention	HighCash	0.0020	0.0014
Intention	LowCash	0.0002	0.0002
Reported	Health	0.5802	0.5503
Reported	HighCash	0.3787	0.2958
Reported	LowCash	0.0682	0.0542
Actual	Health	0.3165	0.2932
Actual	HighCash	0.6469	0.6033
Actual	LowCash	0.0191	0.0186

Supplementary Table S4: Verified Vaccination Regression: Robust to District Samples.

	Model1	Model2	Model3	Model4	Model5	Model6
High Cash	0.96 (0.44, 2.09)	1.03 (0.39, 2.71)	1.06 (0.49, 2.30)	0.70 (0.26, 1.88)	0.94 (0.42, 2.11)	1.07 (0.48, 2.40)
Low Cash	2.04 (1.15, 3.61)	1.80 (0.96, 3.36)	2.38 (1.21, 4.67)	1.47 (0.75, 2.86)	2.15 (1.19, 3.90)	2.16 (1.17, 3.97)
Health	0.68 (0.33, 1.41)	0.65 (0.26, 1.61)	0.73 (0.36, 1.49)	0.80 (0.39, 1.63)	0.69 (0.32, 1.45)	0.61 (0.26, 1.43)
Access	1.04 (0.96, 1.13)	1.02 (0.94, 1.12)	1.02 (0.91, 1.14)	1.10 (1.03, 1.18)	1.05 (0.96, 1.14)	1.05 (0.95, 1.15)
Age	1.00 (1.00, 1.01)	1.00 (0.99, 1.01)	1.00 (1.00, 1.01)	1.00 (0.99, 1.01)	1.00 (1.00, 1.01)	1.01 (1.00, 1.02)
Male	0.98 (0.75, 1.29)	1.08 (0.79, 1.48)	1.05 (0.77, 1.43)	0.73 (0.54, 0.99)	1.00 (0.76, 1.32)	0.98 (0.74, 1.30)
High-Educate	2.00 (0.82, 4.90)	1.87 (0.66, 5.30)	2.54 (0.95, 6.80)	1.23 (0.38, 3.98)	1.96 (0.79, 4.83)	2.32 (0.95, 5.69)
Medium-Educate	1.88 (1.25, 2.83)	1.80 (1.11, 2.90)	1.78 (1.14, 2.78)	1.71 (1.02, 2.87)	1.81 (1.20, 2.73)	1.99 (1.29, 3.06)
Low-Educate	1.38 (0.95, 2.00)	1.15 (0.73, 1.82)	1.25 (0.84, 1.85)	1.34 (0.87, 2.08)	1.40 (0.97, 2.02)	1.43 (0.96, 2.13)
Employed	1.14 (0.89, 1.47)	1.16 (0.86, 1.58)	1.15 (0.86, 1.53)	1.18 (0.90, 1.55)	1.11 (0.86, 1.44)	1.11 (0.85, 1.44)
Mean-Food	1.00 (1.00, 1.00)	1.00 (1.00, 1.00)	1.00 (1.00, 1.00)	1.00 (1.00, 1.00)	1.00 (1.00, 1.00)	1.00 (1.00, 1.00)
Social Media	1.01 (0.99, 1.02)	1.00 (0.99, 1.02)	1.01 (0.99, 1.02)	1.01 (1.00, 1.03)	1.01 (0.99, 1.02)	1.00 (0.99, 1.02)
District 2			0.03 (0.01, 0.10)	0.04 (0.01, 0.11)	0.03 (0.01, 0.10)	0.03 (0.01, 0.10)
District 3	1.22 (0.62, 2.39)	0.04 (0.01, 0.11)		0.04 (0.02, 0.10)	0.04 (0.02, 0.11)	0.04 (0.02, 0.11)
District 4	3.20 (1.39, 7.34)	0.10 (0.04, 0.31)	0.11 (0.04, 0.33)		0.11 (0.04, 0.32)	0.11 (0.04, 0.32)
District 5	0.17 (0.07, 0.46)	0.01 (0.00, 0.02)	0.01 (0.00, 0.02)	0.01 (0.00, 0.02)		0.01 (0.00, 0.02)
District 6	4.10 (1.84, 9.15)	0.14 (0.05, 0.41)	0.15 (0.05, 0.45)	0.12 (0.04, 0.32)	0.13 (0.05, 0.39)	
Intercept	0.10 (0.04, 0.23)	5.21 (1.50, 18.06)	3.18 (0.93, 10.89)	2.51 (0.82, 7.63)	2.93 (0.94, 9.14)	2.41 (0.74, 7.88)
Observations	2105	1522	1723	1553	1800	2026
Akaike Inf. Crit.	2194.68	1610.09	1749.89	1422.33	2100.52	2052.53
Log Likelihood	-1080.34	-788.04	-857.94	-694.17	-1033.26	-1009.26

Supplementary Table S5: Spillover Regression Estimates Including Population Size Interactions.

	Model1	Model2	ModelSP1	ModelSP2
CDC_placebo_SizeLarge	0.67 (0.22, 2.03)	4.28 (0.60, 30.65)		
CDC_Placebo_SizeMid	0.69 (0.23, 2.12)	3.54 (0.52, 24.36)		
CDC_SizeLarge	0.76 (0.39, 1.50)	2.95 (0.37, 23.28)	3.50 (1.19, 10.25)	1.68 (0.16, 17.47)
CDC_SizeMid	0.76 (0.37, 1.57)	2.59 (0.27, 25.36)	2.07 (0.66, 6.48)	1.67 (0.14, 19.19)
Health	1.34 (0.74, 2.43)	0.32 (0.06, 1.61)		
Health_Placebo	1.15 (0.44, 2.98)	0.29 (0.08, 1.06)		
HighCash	1.63 (0.87, 3.08)	0.65 (0.12, 3.57)		
HighCash_Placebo	1.55 (0.72, 3.35)	0.53 (0.13, 2.05)		
HighCash_placebo_SizeLarge	0.90 (0.34, 2.36)	2.46 (0.38, 16.07)		
HighCash_placebo_SizeMid	0.51 (0.20, 1.30)	1.92 (0.27, 13.73)		
HighCash_SizeLarge	0.63 (0.31, 1.30)	1.91 (0.23, 15.91)	0.95 (0.33, 2.77)	0.92 (0.10, 8.22)
HighCash_SizeMid	0.58 (0.27, 1.23)	1.33 (0.09, 19.93)	1.17 (0.38, 3.61)	0.88 (0.07, 10.80)
LowCash	1.40 (0.75, 2.61)	0.85 (0.18, 3.98)		
LowCash_Placebo	0.71 (0.33, 1.51)	0.83 (0.17, 4.05)		
LowCash_Placebo_SizeLarge	1.28 (0.53, 3.06)	4.43 (0.66, 29.88)		
LowCash_Placebo_SizeMid	1.86 (0.75, 4.61)	1.24 (0.16, 9.50)		
LowCash_SizeLarge	0.89 (0.44, 1.78)	4.25 (0.62, 28.94)	2.19 (0.67, 7.11)	3.22 (0.37, 27.68)
LowCash_SizeMid	0.98 (0.46, 2.08)	1.48 (0.18, 11.95)	1.01 (0.29, 3.48)	1.18 (0.09, 16.45)
VCash_Health			0.48 (0.21, 1.08)	0.27 (0.04, 1.67)
VCash_HighCash			1.15 (0.49, 2.68)	0.87 (0.16, 4.74)
VCash_LowCash			0.74 (0.30, 1.85)	0.62 (0.09, 4.33)
Village_Population_SizeLarge	1.51 (0.88, 2.57)	0.15 (0.04, 0.57)	0.63 (0.27, 1.49)	0.14 (0.03, 0.69)
Village_Population_SizeMid	1.34 (0.75, 2.38)	0.25 (0.06, 1.10)	0.59 (0.24, 1.48)	0.24 (0.03, 1.71)
Intercept	0.53 (0.31, 0.90)	21.78 (4.90, 96.68)	1.15 (0.44, 2.96)	11.82 (0.55, 254.78)
Covariates	Yes	Yes	Yes	Yes
Fixed Effects	Yes	Yes	Yes	Yes
Observations	3957	2146	1035	499
Akaike Inf. Crit.	5183.65	2187.74	1304.94	530.63
Log Likelihood	-2556.82	-1058.87	-626.47	-239.31

Ghana Vaccine Incentives Phase I Survey

Start of Block: Enumerator Covid Survey

Q147 **Enumerators**, please **answer** the following COVID-19 assessment questions **BEFORE** starting the interview.

Page Break

Q166 Have you had at least one COVID-19 vaccine?

○ Yes (1)

○ No (2)

Q131 Have you experienced a fever of 100.4F (38C) or greater, a new cough, new loss of taste or smell, or a shortness of breath within the past 14 days?

○ Yes (1)

○ No (2)

Q132 In the past 14 days, have you been tested positive for COVID-19?

○ Yes (1)

○ No (2)

Q133 To the best of your knowledge, in the past 14 days, have you been in close contact (within 6 feet (1.5 meter) or less for at least 10 minutes) with anyone while they had COVID-19?

○ Yes (1)

○ No (2)

Q134 In the past 14 days, have you traveled to a place that is having widespread community transmission of COVID-19?

○ Yes (1)

○ No (2)

End of Block: Enumerator Covid Survey

Start of Block: Enumerator survey

E1 Please enter your device id

____

E2 Please enter your enumerator id

____

E3 Please enter the dwelling sequence number (Number of dwellings you passed by since the previous interview)

____

Q123 Please enter the village id

____

E4 Did you identify any eligible respondents in this household?

○ Yes (1)

○ No (2)

End of Block: Enumerator survey

Start of Block: Skip Household Questions

Q107 Please Enter the Contact information for this household

○ There was no answer at this household (1)

○ There was no one 18 years or over in the household (2)

○ All household members refused to participate (3)

End of Block: Skip Household Questions

Start of Block: Enumerator ID Survey

*

E5 Please describe the random selection of respondent from household members

____

Page Break

E6 Respondent's First name (Respondent's first name as it appears on their Ghana ID Card.)

____

E7 Respondent's Last name (Respondent's last name as it appears on their Ghana ID Card.)

____

End of Block: Enumerator ID Survey

Start of Block: COVID-19 consent

Q135 Your household was selected as one of those to which the survey questions will be asked. You were not selected for any specific reason. Rather, your household was selected randomly from a list of all the households in this village/city. All the information that your household provides is strictly confidential. It will not be shared. Please spare some time to answer the questions.   All field staff are required to wear masks throughout the interview, sanitize their hands and equipment before the interview and check their temperature in the morning. All field staff have had a COVID-19 test within the past 2 days.   The interview will only take place in an open space where the field staff can maintain a distance of 1.5 meters from all members of your household. You will be required to wear a face mask during the interview, and we will provide you with a face make and disinfectant gel before the interview begins. However, given the nature of the virus, there is an inherent risk of becoming infected with COVID-19 by proceeding with this interview.   Should any of the field staff that work with you today become sick from COVID-19, we will inform you as soon as possible. And if you have any queries or COVID-19 related information to provide us please contact us at the number we have provided you. We thank you in advance for your time. Do you consent to take part in the survey?

- ○ Yes, I consent and will take part in the survey. (4)
- ○ No, I do NOT consent and therefore will NOT take part in the survey (5)

End of Block: COVID-19 consent

Start of Block: Household Covid Survey

Q127 Have you experienced a fever of 100.4F (38C) or greater, a new cough, new loss of taste or smell, or a shortness of breath within the past 14 days?

- Yes (1)
- No (2)

Q128 In the past 14 days, have you been tested positive for COVID-19?

- Yes (2)
- No (3)

Q129 To the best of your knowledge, in the past 14 days, have you been in close contact (within 6 feet (1.5 meter) or less for at least 10 minutes) with anyone while they had COVID-19?

- Yes (1)
- No (2)

Q130 In the past 14 days, have you traveled to a place that is having widespread community transmission of COVID-19?

- Yes (1)
- No (2)

End of Block: Household Covid Survey

Start of Block: Questions to enumerator about COVID 19

Q140 Has the interviewee agreed to: Wear a mask Social distancing

○ Yes (1)

○ No (2)

End of Block: Questions to enumerator about COVID 19

Start of Block: Consent

Q1.1 We are researchers from the University of Ghana and the University of Oxford, United Kingdom, conducting a study on COVID-19 vaccines. The project is led by Dr. Edward Asiedu of the University of Ghana, working in collaboration with Professor Philip Clarke and Professor Raymond Duch from the University of Oxford, and their team of researchers to better understand vaccine uptake. We very much appreciate your interest in participating in this study. Please carefully consider this information before agreeing to participate by ticking the 'yes' box below. You will be asked some questions about yourself. This should take about 5 minutes. No background knowledge is required. The information that we gather from this study will be used to understand more about COVID-19 vaccinations.

Do I have to take part? Your participation is voluntary. You may withdraw at any point during the questionnaire for any reason before we submit your answers – just indicate to the enumerator that you would like to withdraw. However, we are only able to reimburse participants who complete the full survey (which will include watching a very short video).

How will my data be used? We will not collect any information that would directly identify a participant, so none of the researchers will be able to identify you. The anonymous survey data that you provide will be made available to researchers and may be used in academic publications. Such data will be stored in a secure cloud (eg. OneDrive for Business) of the University of Oxford for a minimum of three years after publication or public release. Anonymous survey data may be securely transferred to, stored and/or processed at a destination outside your country of residence. By completing the interview, you agree to the collection, transfer, storage and processing of your data. This transfer, storage or processing of data will be conducted in such a way that no one will be able to identify you.

Who will have access to my data? The University of Ghana and the University of Oxford will have access to your survey data. The University will process your data for the purpose of the research outlined above. Research is a task that we perform in the public interest. Further information about your rights with respect to your personal data is available from https://compliance.admin.ox.ac.uk/individual-rights. Responsible members of the University of Oxford and funders may be given access to your survey data for monitoring and/or audit of the study to ensure we are complying with guidelines, or as otherwise required by law. Can I opt-out from this

panel or ask for my data to be deleted? Participants cannot withdraw themselves or their data once they have submitted the survey responses. We will retain a copy of the data on our, University of Ghana and University of Oxford, servers. This survey is for a research project of the Health Economics Research Centre based at the University of Oxford and the Experimental Development Economics Group at the University of Ghana. The Principal

Researcher is Professor Philip Clarke, who is the Director of the Health Economics Research Centre at the University of Oxford. The Co-Principal Researchers are Dr. Edward Asiedu, who is a development Economist at the University of Ghana and Professor Raymond Duch, who is the Director of the Centre of Experimental Social Sciences at the University of Oxford. This project has been reviewed by, and received ethics clearance through, the University of Oxford Central University Research Ethics Committee.

Payment. You will receive payment of 5GHS in cash after your participation in today's study is finished. You will be paid immediately. We will follow up and contact you again in 6 weeks – you will receive another 5 GHS when we contact you.

Who do I contact if I have a concern about the study or I wish to complain? If you have a concern about any aspect of this study, please contact Dr. Edward Asiedu at the University of Ghana, email edasiedu@ug.edu.gh and phone at 0550244204 and Professor Raymond Duch at the University of Oxford, email raymond.duch@nuffield.ox.ac.uk and phone number +44 (0)1865 278 515. We will acknowledge your concern within 10 working days and give you an indication of how it will be dealt with. If you remain unhappy or wish to make a formal complaint, please contact the Chair of the Research Ethics Committee at the University of Oxford who will seek to resolve the matter as soon as possible: Medical Sciences Interdivisional Research Ethics Committee (MS IDREC). Research Services, University of Oxford, Wellington Square, Oxford, OX1 2JD Email: ethics@medsci.ox.ac.uk Please note that you may only participate in this survey if you are 18 years of age or over. If you have considered the information we have just presented to you and agree to participate with the understanding that the data (including any personal data) you submit will be processed accordingly, please check the relevant box below to get started.

Q1.2 Please note that you may only participate in this survey if you are **18 years of age or over**.

○ Yes, I certify that I am 18 years of age or over (1)

○ No (2)

Q1.3 If you have read the information above and agree to participate with the understanding that the data (including any personal data) you submit will be processed accordingly, please check the relevant box below to get started.

○ Yes, I agree to take part (1) ○ No (2)

End of Block: Consent

Start of Block: Vaccine Question

Q3.1 Have you had at least one of the COVID-19 Vaccine shots?

○ Yes, I have (1)

○ No, I haven't (2)

End of Block: Vaccine Question

Start of Block: Demographics 1

Q2.1

Thank you for agreeing to participate in our short survey. We would like to ask you some basic questions about yourself. This information is being collected only for statistical purposes. We will ask you a small number of these questions. A short video with some information about COVID-19 vaccines will end the survey.

Page Break

*

Q2.2 What is your current age?

____

Q2.3 What is your gender?

○ Male (1)

○ Female (2)

○ Other (4) ____

○ Prefer not to say (5)

Page Break

Q2.4 Select the region and district you live in.

Region (1)

District (2)

▼ Ahafo (1) ... Western North ~ Suaman District (276)

Page Break

*

Q141 How many people live in the house together with you (NOT including you) at this moment?

____

*

Q142 How many children below 18 years old are currently living in your home?

____

Q143 What is your current working situation?

- ◯ Employed (full time) (1)
- ◯ Employed (part time) (2)
- ◯ Unemployed (3)
- ◯ Retired (4)
- ◯ Student (5)
- ◯ Home maker (6)

*

Q144 How much on average does your household spend in a typical week on food?

____

*

Q145 How much on average does your household spend in a typical week on non-food items (electricity, water, rent, school fees)?

____

Q146 How would you rate the overall economic or financial condition of your household today?

- ○ Very good (1)
- ○ Good (2)
- ○ Neither good nor bad (3)
- ○ Bad (4)
- ○ Very bad (5)

Q2.5 What is the highest educational qualification you have completed?

- ○ Never attended (5)
- ○ Nursery (4)
- ○ Kindergarten (9)
- ○ Primary (10)
- ○ JSS/JHS (15)
- ○ Middle (11)
- ○ SSS/SHS (38)
- ○ Vocational/Technical/Commercial (40)
- ○ Post middle/secondary certificate (41)
- ○ Post-secondary diploma (42)
- ○ Bachelor degree (43)
- ○ Post graduate (Cert. Diploma Masters PHD etc) (45)

End of Block: Demographics 1

Start of Block: Vaccine Incentives Section (Placebo 0.25)

Q5.1 Please click on the play arrow for this video. **You have to watch the full video in order to continue the survey.**

Page Break

Q101 Do you think you will get a first shot of a COVID-19 vaccine within the first 6 weeks after the vaccine becomes available to you?

○ Yes (1) ○ No (2) ○ Do not know (3)

○ Prefer not to say (4)

Display This Question:

If Do you think you will get a first shot of a COVID-19 vaccine within the first 6 weeks after the v... = No

Q154 Why will you NOT get vaccinated against COVID-19? (check all that apply)

▢ I don't believe the COVID-19 vaccine will be effective (1)

▢ I am a pregnant/nursing mother and cannot be vaccinated (2)

▢ I cannot be vaccinated due to medical reasons as advised by a doctor (3)

▢ I am concerned about dangerous side effects from the COVID-19 vaccine (4)

▢ Enough other people will accept vaccination so i will benefit from herd immunity (5)

▢ I have already been infected with COVID-19 and believe I have developed natural immunity (6)

- The COVID-19 virus will not be very harmful to my health (7)
- I don't trust the health care providers in this country (8)
- Other (9)

Q102 We understand that there is always some uncertainty regarding all decisions. From 0% to 100%, what do you think are the chances that you will choose to get a first shot of a COVID-19 vaccine within the first 6 weeks after the vaccine becomes available to you?

	0 10 20 30 40 50 60 70 80 90 100
4 ()	

Q157 Do you currently use any solar-panel devices to charge your electrical appliances such as mobile phones or house appliances?

- Yes (1)
- No (2)
- Don't know

(4)

Q162 How likely would you consider buying a solar-panel device to charge your electrical appliances such as mobile phones or house appliances. From 0% to 100%, what do you think are the chances that you will buy a solar-panel device?

0 10 20 30 40 50 60 70 80 90 100

4 ()	

Q104 Thank you for participating in our study.

End of Block: Vaccine Incentives Section (Placebo 0.25)

Start of Block: Payment Survey

P1 We need some additional information for the payment. Please, complete the following information.

Q91 Do you have a mobile number where you can get paid?

○ Yes, I have a number registered (1)

○ No, I do not have a mobile number where I can get paid (2)

Q92 First Name

____

Q93 Last Name

____

Q94 Mobile Number

____

Q95 Confirm your Mobile Number

____

End of Block: Payment Survey

Start of Block: Payment Relative

Display This Question:

If Do you have a mobile number where you can get paid? = No, I do not have a mobile number where I can get paid

Q148 Is there a mobile number of a family member or friend that we can contact you on?

- Yes (1)
- No (2)

Q150 Mobile Number

____

Q151 Confirm your Mobile Number

____

End of Block: Payment Relative

Start of Block: Vaccine Incentives Section (CDC Health Control)

Q4.1 Please click on the play arrow for this video. **You have to watch the full video in order to continue the survey.**

Page Break

Q109 Do you think you will get a first shot of a COVID-19 vaccine within the first 6 weeks after the vaccine becomes available to you?

◯ Yes (1) ◯ No (2) ◯ Do not know (3)

◯ Prefer not to say (4)

Display This Question:

If Do you think you will get a first shot of a COVID-19 vaccine within the first 6 weeks after the v... = No

Q152 Why will you NOT get vaccinated against COVID-19? (check all that apply)

▢ I don't believe the COVID-19 vaccine will be effective (1)

▢ I am a pregnant/nursing mother and cannot be vaccinated (2)

▢ I cannot be vaccinated due to medical reasons as advised by a doctor (3)

▢ I am concerned about dangerous side effects from the COVID-19 vaccine (4)

☐ Enough other people will accept vaccination so i will benefit from herd immunity (5)

☐ I have already been infected with COVID-19 and believe i have developed natural immunity (6)

☐ The COVID-19 virus will not be very harmful to my health (7)

☐ I don't trust the health care providers in this country (8)

☐ Other (9)

Q110 We understand that there is always some uncertainty regarding all decisions. From 0% to 100%, what do you think are the chances that you will choose to get a first shot of a COVID-19 vaccine within the first 6 weeks after the vaccine becomes available to you?

	0 10 20 30 40 50 60 70 80 90 100
4 ()	

Q153 Do you currently use any solar-panel devices to charge your electrical appliances such as mobile phones or house appliances?

○ Yes (1)

○ No (2)

○ Don't know (4)

Q165 How likely would you consider buying a solar-panel device to charge your electrical appliances such as mobile phones or house appliances. From 0% to 100%, what do you think are the chances that you will buy a solar-panel device?

	0 10 20 30 40 50 60 70 80 90 100
4 ()	

Page Break

Q103 Thank you for participating in our study.

We indicated that we will follow up with you in 6 weeks. We will contact you in order to verify your vaccination status. If you can provide us with your COVID-19 vaccination carnet at the time, we will upload a copy of the vaccination carnet to our secure server for verification.

End of Block: Vaccine Incentives Section (CDC Health Control)

Start of Block: Vaccine Incentives Section (Low Cash)

Q159 Please click on the play arrow for this video. **You have to watch the full video in order to continue the survey.**

Page Break

Q160 Do you think you will get a first shot of a COVID-19 vaccine within the first 6 weeks after the vaccine becomes available to you?

○ Yes (1) ○ No (2) ○ Do not know (3)

○ Prefer not to say (4)

Display This Question:

If Do you think you will get a first shot of a COVID-19 vaccine within the first 6 weeks after the v... = No

Q155 Why will you NOT get vaccinated against COVID-19? (check all that apply)

▢ I don't believe the COVID-19 vaccine will be effective (1)

▢ I am a pregnant/nursing mother and cannot be vaccinated (2)

▢ I cannot be vaccinated due to medical reasons as advised by a doctor (3)

▢ I am concerned about dangerous side effects from the COVID-19 vaccine (4)

▢ Enough other people will accept vaccination so i will benefit from herd immunity (5)

▢ I have already been infected with COVID-19 and believe i have developed natural immunity (6)

▢ The COVID-19 virus will not be very harmful to my health (7)

☐ I don't trust the health care providers in this country (8)

☐ Other (9)

Q161 We understand that there is always some uncertainty regarding all decisions. From 0% to 100%, what do you think are the chances that you will choose to get a first shot of a COVID-19 vaccine within the first 6 weeks after the vaccine becomes available to you?

	0 10 20 30 40 50 60 70 80 90 100
4 ()	

Q158 Do you currently use any solar-panel devices to charge your electrical appliances such as mobile phones or house appliances?

○ Yes (1)

○ No (2)

○ Don't know

(4)

Q163 How likely would you consider buying a solar-panel device to charge your electrical appliances such as mobile phones or house appliances. From 0% to 100%, what do you think are the chances that you will buy a solar-panel device?

0 10 20 30 40 50 60 70 80 90 100

4 ()	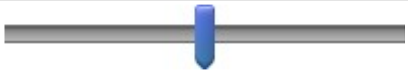

Page Break

Q162 Thank you for participating in our study.

We indicated that we will follow up with you in 30 days. We will contact you in order to verify your vaccination status. If you can provide us with your COVID-19 vaccination carnet at the time, we will upload a copy of the vaccination carnet to our secure server for verification and you will be paid your 3 USD via cell phone money payment or by cash if you prefer.

End of Block: Vaccine Incentives Section (Low Cash)

Start of Block: Vaccine Incentives Section (High Cash)

Q163 Please click on the play arrow for this video. **You have to watch the full video in order to continue the survey.**

Page Break

Q164 Do you think you will get a first shot of a COVID-19 vaccine within the first 6 weeks after the vaccine becomes available to you?

○ Yes (1) ○ No (2) ○ Do not know (3)

○ Prefer not to say (4)

Display This Question:

If Do you think you will get a first shot of a COVID-19 vaccine within the first 6 weeks after the v... = No

Q156 Why will you NOT get vaccinated against COVID-19? (check all that apply)

▢ I don't believe the COVID-19 vaccine will be effective (1)

▢ I am a pregnant/nursing mother and cannot be vaccinated (2)

▢ I cannot be vaccinated due to medical reasons as advised by a doctor (3)

▢ I am concerned about dangerous side effects from the COVID-19 vaccine (4)

▢ Enough other people will accept vaccination so i will benefit from herd immunity (5)

▢ I have already been infected with COVID-19 and believe i have developed natural immunity (6)

▢ The COVID-19 virus will not be very harmful to my health (7)

☐ I don't trust the health care providers in this country (8)

☐ Other (9)

Q165 We understand that there is always some uncertainty regarding all decisions. From 0% to 100%, what do you think are the chances that you will choose to get a first shot of a COVID-19 vaccine within the first 6 weeks after the vaccine becomes available to you?

	0 10 20 30 40 50 60 70 80 90 100
4 ()	

Q159 Do you currently use any solar-panel devices to charge your electrical appliances such as mobile phones or house appliances?

○ Yes (1)

○ No (2)

○ Don't know

(4)

Q161 How likely would you consider buying a solar-panel device to charge your electrical appliances such as mobile phones or house appliances. From 0% to 100%, what do you think are the chances that you will buy a solar-panel device?

	0 10 20 30 40 50 60 70 80 90 100
4 ()	

Page Break

Q166 Thank you for participating in our study.

We indicated that we will follow up with you in 6 weeks. We will contact you in order to verify your vaccination status. If you can provide us with your COVID-19 vaccination carnet at the time, we will upload a copy of the vaccination carnet to our secure server for verification and you will be paid your 10 USD via cell phone money payment or by cash if you prefer.

End of Block: Vaccine Incentives Section (High Cash)

Phase II: Ghana Incentives Post Treatment Survey

Start of Block: Enumerator ID

Q1.1 Please enter your enumerator ID (Only integers)

____

End of Block: Enumerator ID

Start of Block: Number to call

Q1.2 Please read the following instructions:

- The link below contains the cellphone number from a participant of the ghana survey. - Follow the instructions to call the number given in the link and dial that number at least 3 times.
- If the subject answers the call or don't answer after 3 tries go to the next slide and solve the question.

Full Name: ${e://Field/name}${e://Field/number}">Click here to call the number in the device

End of Block: Number to call

Start of Block: Did the respondent answer the phone?

Q2.1 Did the respondent answer the phone?

- Yes (1)
- No (2)

○ Contact switched off or cannot be reached (3)

○ Contact and number did not match (wrong contact) (4)

Page Break

Display This Question:

If Did the respondent answer the phone? = Yes

Q3.1 We are texting you from the CANDOUR project. Can you answer some quick questions and you will receive 6 CEDI via your xxxxxx?

- ◯ Yes, I agree (1)
- ◯ No (2)

End of Block: Did the respondent answer the phone?

Start of Block: Interview Satisfaction

Q6.1 Were you satisfied with the interview?

◯ Yes (1) ◯ No (2)

◯ Was not interviewed (3)

End of Block: Interview Satisfaction

Start of Block: Topic

Q7.1 What was the topic of the interview?

- ◯ COVID-19 Vaccines (1)
- ◯ Transportation to you village (3)
- ◯ Schools in your village (4)

End of Block: Topic

Start of Block: Have you received a COVID-19 vaccine?

Q8.1 Have you received a COVID-19 vaccine?

- ○ Yes, I have received a COVID-19 vaccine (1)
- ○ No, I have not received a COVID-19 vaccine (2)

Display This Question:

If Have you received a COVID-19 vaccine? = Yes, I have received a COVID-19 vaccine

*

Q8.2 Please enter the date you have received your COVID-19 vaccine? dd/mm/yyyy

____

Display This Question:

If Have you received a COVID-19 vaccine? = Yes, I have received a COVID-19 vaccine

Q8.3 Why did you decide to get vaccinated against COVID-19, what were the reasons? (check all that apply)

- ☐ To protect myself (1)
- ☐ To protect my family (2)
- ☐ To be able to travel (3)
- ☐ To be able to visit family and friends (4)
- ☐ To be able to enter public venues like restaurants, cinemas and concerts (5)
- ☐ To protect the public (people I don't know) (6)
- ☐ Because everyone else will (7)
- ☐ Because friends and family recommend it (8)
- ☐ Because doctors recommend it (9)
- ☐ Because health officials recommend it (10)
- ☐ Because politicians recommend it (11)
- ☐ Because of contact with COVID-19 infected people (12)

☐ Because I had COVID-19 related symptoms (13)

☐ Because it was required for my work or school (14)

☐ Other (15) ____

End of Block: Have you received a COVID-19 vaccine?

Start of Block: Spouse and Partner

Q5.1 Do you live with a spouse or partner?

○ Yes (1)

○ No (2)

Page Break

Display This Question:

If Do you live with a spouse or partner? = Yes

Q5.2 Did your spouse or partner get a COVID-19 vaccine?

○ Yes (1)

○ No (2)

Display This Question:

If Did your spouse or partner get a COVID-19 vaccine? = Yes

*

Q5.3 When did your spouse or partner get a COVID-19 vaccine? dd/mm/yyy

____

End of Block: Spouse and Partner

Start of Block: Reason NOT to GET the COVID-19 vaccine

Display This Question:

If Have you received a COVID-19 vaccine? = No, I have not received a COVID-19 vaccine

Q9.1 Why did you decide NOT to get vaccinated against COVID-19, what are / were reasons?

(check all that apply)

▢ I don't believe the COVID-19 vaccine will be effective (1)

▢ I cannot be vaccinated due to medical reasons as advised by a doctor (2)

▢ I am concerned about dangerous side effects from the COVID-19 vaccine (3)

☐ Enough other people will accept vaccination so I will benefit from herd immunity (4)

☐ I have already been infected with COVID-19 and believe I have developed natural immunity (5)

☐ The COVID-19 virus will not be very harmful to my health (6)

☐ I don't trust the health care providers in this country (7)

☐ I am pregnant or i am about to give birth (9)

☐ Other (8) ____

End of Block: Reason NOT to GET the COVID-19 vaccine

Start of Block: 10

Q10.1 Have you recently purchased a solar charging device?

○ Yes, I have recently purchased a solar charging device (1)

○ No, I have not recently purchased a solar charging device (2)

Display This Question:

If Have you recently purchased a solar charging device? = Yes, I have recently purchased a solar charging device

*

Q10.2 Please enter the date you have purchased a solar charging device? dd/mm/yyyy

____

End of Block: 10

Start of Block: Block 12

*

Q25 How many villages in the district do you think you have visited in the last month?

____

---

*

Q26 How many villages in the district do you think you have visited in the last year?

____

Q27 Do you have family in other villages in the district?

- ○ Yes (1)
- ○ No (2)

---

*

Q28 How many family members do you have in another village?

____

Q29 Do you have friends and acquaintances who are not part of your family who live in other villages in the district?

◯ Yes (1)

◯ No (2)

Q30 How many friends and acquaintances who are not part of your family do you have in another village?

____

End of Block: Block 12

Start of Block: Network Densitiy Question

Q4.1 How many individuals can you identify in your social network? Think of friends and relatives that live close to you

____

End of Block: Network Densitiy Question

Start of Block: Whatsapp

Q2.2 Do you have WhatsApp?

◯ Yes (1)

◯ No (2)

Q2.3 How often do you use WhatsApp?

- ○ Many times, every day (1)
- ○ A few times every day (4)
- ○ About once a day (5)
- ○ A few times each week (6)
- ○ About once a week (7)
- ○ Once or twice a month (8)
- ○ Less than once a month (9)
- ○ Never (10)

Q2.5 What social media have you used in the last year?

- ▢ Facebook (1)
- ▢ Twitter (4)
- ▢ Instagram (5)
- ▢ Reddit (6)

- [ ] YouTube (7)
- [ ] SnapChat (8)
- [ ] TikTok (9)
- [ ] Other (10)
- [ ] I don’t use social media (11)

Q2.4 You have WhatsApp groups with...

- [ ] Close family (parents, children and siblings) (1)
- [ ] Close friends (4)
- [ ] College, university or work colleagues (5)
- [ ] Far family (cousins, uncles, nephews)Neighbors (6)
- [ ] Church groups (7)
- [ ] Other groups (8)
- [ ] I don't have groups (9)

Q2.6 How often do you use social media?

○ Many times, every day (1)

○ A few times every day (4)

○ About once a day (5)

○ A few times each week (6)

○ About once a week (7)

○ Once or twice a month (8)

○ Less than once a month (9)

○ Never (10)

End of Block: Whatsapp

Start of Block: Payment survey

Q11.1 How would you like to receive your recompensation for taking the survey?

○ I would like to receive my recompensation in mobile money (1)

○ I would like to receive my recompensation in person (NOTE: This will take 6 weeks to process) (2)

○ No (3)

End of Block: Payment survey

Start of Block: Block 12

Q12.1 You will receive $${e://Field/payment} for your participation in our COVO-19 Vaccine study.

End of Block: Block 12

Phase III: In-person Post-treatment Survey

Start of Block: Enumerator survey

Q66 **MOP-UP SURVEY -- FOLLOW UP TO BASELINE TREATMENT**

Q1 Please enter your device id

____

Q2 Please enter your enumerator id

____

---

*

Q4 Please enter the village id

____

Q5 Did you identify the eligible respondents in this household?

○ Yes (1) ○ No (2) ○ There was no answer at this household (4)

End of Block: Enumerator survey

Start of Block: Enumerator ID Survey

Page Break

Q2 Respondent's First name (Respondent's first name as it appears on their Ghana ID Card.)

____

Q3 Respondent's Last name (Respondent's last name as it appears on their Ghana ID Card.)

____

End of Block: Enumerator ID Survey

Start of Block: CANDOUR consent

Page Break

Q3.1 We visited your household some months ago and showed you a short video. We would like to ask you some brief additional questions. Can you answer some quick questions and you will receive 5 CEDI in cash?

- ○ Yes, I agree (1)
- ○ No (2)

End of Block: CANDOUR consent

Start of Block: Interview Satisfaction

Q6.1 Were you satisfied with the interview we conducted with you a couple months ago?

- ○ Yes (1)
- ○ No (2)
- ○ Was not interviewed (3)

End of Block: Interview Satisfaction

Start of Block: Topic

Q7.1 What was the topic of the interview?

- ○ COVID-19 Vaccines (1)
- ○ Transportation to your village (3)
- ○ Schools in your village (4)

End of Block: Topic

Start of Block: Have you received a COVID-19 vaccine?

Q8.1 Have you received at least one shot of a COVID-19 vaccine?

○ Yes, I have received at least one shot of a COVID-19 vaccine (1)

○ No, I have not received at least one shot of a COVID-19 vaccine (2)

Display This Question:

If Have you received at least one shot of a COVID-19 vaccine? = Yes, I have received at least one shot of a COVID-19 vaccine

*

Q8.2 Please enter the date you have received your COVID-19 vaccine? dd/mm/yyyy

____

Display This Question:

If Have you received at least one shot of a COVID-19 vaccine? = Yes, I have received at least one shot of a COVID-19 vaccine

Q8.3 Why did you decide to get vaccinated against COVID-19, what were the reasons? (check all that apply)

- ☐ To protect myself (1)
- ☐ To protect my family (2)
- ☐ To be able to travel (3)
- ☐ To be able to visit family and friends (4)
- ☐ To be able to enter public venues like restaurants, cinemas and concerts (5)
- ☐ To protect the public (people I don't know) (6)
- ☐ Because everyone else will (7)
- ☐ Because friends and family recommend it (8)
- ☐ Because doctors recommend it (9)
- ☐ Because health officials recommend it (10)
- ☐ Because politicians recommend it (11)
- ☐ Because of contact with COVID-19 infected people (12)

☐ Because I had COVID-19 related symptoms (13)

☐ Because it was required for my work or school (14)

☐ Other (15) ____

Display This Question:

If Have you received at least one shot of a COVID-19 vaccine? = Yes, I have received at least one shot of a COVID-19 vaccine

Q64 Have you received a second COVID-19 vaccine shot?

○ Yes, I have received a second COVID-19 vaccine shot (1)

○ No, I have not received a second COVID-19 vaccine shot (2)

Display This Question:

If Have you received a second COVID-19 vaccine shot? = Yes, I have received a second COVID-19 vaccine shot

*

Q65 Please enter the date you have received your second COVID-19 vaccine? dd/mm/yyyy

____

Display This Question:

If Have you received at least one shot of a COVID-19 vaccine? = Yes, I have received at least one shot of a COVID-19 vaccine

Q55 **FOR ENUMERATOR:** Did you review the respondent's vaccination card?

○ Yes (1)

○ No (2)

End of Block: Have you received a COVID-19 vaccine?

Start of Block: Spouse and Partner

Q5.1 Do you live with a spouse or partner?

○ Yes (1)

○ No (2)

Page Break

Display This Question:

If Do you live with a spouse or partner? = Yes

Q5.2 Did your spouse or partner get a COVID-19 vaccine?

○ Yes (1)

○ No (2)

Display This Question:

If Did your spouse or partner get a COVID-19 vaccine? = Yes

Q56 FOR ENUMERATOR: Did you review the spouse or partner's vaccination card?

○ Yes (1)

○ No (2)

Display This Question:

If Did your spouse or partner get a COVID-19 vaccine? = Yes

Q5.3 When did your spouse or partner get a COVID-19 vaccine? dd/mm/yyy

____

End of Block: Spouse and Partner

Start of Block: Reason NOT to GET the COVID-19 vaccine

Display This Question:

If Have you received at least one shot of a COVID-19 vaccine? = No, I have not received at least one shot of a COVID-19 vaccine

Q9.1 Why did you decide NOT to get vaccinated against COVID-19, what are / were reasons?

(check all that apply)

▢ There was no vaccine facility in my village. (10)

▢ I don't believe the COVID-19 vaccine will be effective (1)

▢ I cannot be vaccinated due to medical reasons as advised by a doctor (2)

▢ I am concerned about dangerous side effects from the COVID-19 vaccine (3)

▢ Enough other people will accept vaccination so I will benefit from herd immunity (4)

▢ I have already been infected with COVID-19 and believe I have developed natural immunity (5)

▢ The COVID-19 virus will not be very harmful to my health (6)

- [ ] I don't trust the health care providers in this country (7)

- [ ] I am pregnant or I am about to give birth (9)

- [ ] I am allergic to the vaccine (12)

- [ ] I could not afford to take time from my work. (11)

- [ ] Other (8) ____

End of Block: Reason NOT to GET the COVID-19 vaccine

Start of Block: Solar Device

Q10.1 Have you recently purchased a solar charging device since we last spoke with you?

○ Yes, I have recently purchased a solar charging device (1)

○ No, I have not recently purchased a solar charging device (2)

Display This Question:

If Have you recently purchased a solar charging device since we last spoke with you? = Yes, I have recently purchased a solar charging device

Q10.2 Please enter the date you have purchased a solar charging device? dd/mm/yyyy

____

End of Block: Solar Device

Start of Block: Block 12

Q25 How many villages in the district do you think you have visited in the last month?

____

*

Q26 How many villages in the district do you think you have visited in the last year?

____

Q27 Do you have family in other villages in the district?

○ Yes (1)

○ No (2)

*

Q28 How many family members do you have in another village?

____

Q29 Do you have friends and acquaintances who are not part of your family who live in other villages in the district?

○ Yes (1)

○ No (2)

*

Q30 How many friends and acquaintances who are not part of your family do you have in another village?

____

End of Block: Block 12

Start of Block: Network Densitiy Question

*

Q4.1 How many individuals can you identify in your social network? Think of friends and relatives that live close to you

____

End of Block: Network Densitiy Question

Start of Block: Whatsapp

Q2.2 Do you have WhatsApp?

○ Yes (1)

○ No (2)

Display This Question:

If Do you have WhatsApp? = Yes

Q2.3 How often do you use WhatsApp?

○ Many times, every day (1)

○ A few times every day (4)

○ About once a day (5)

A few times each week (6)

About once a week (7)

Once or twice a month (8)

Less than once a month (9)

Never (10)

Display This Question:

If Do you have WhatsApp? = Yes

Q2.4 You have WhatsApp groups with...

Close family (parents, children and siblings) (1)

Close friends (4)

College, university or work colleagues (5)

Far family (cousins, uncles, nephews)Neighbors (6)

Church groups (7)

Other groups (8)

I don't have groups (9)

Q2.5 What social media have you used in the last year?

- ☐ Facebook (1)
- ☐ Twitter (4)
- ☐ Instagram (5)
- ☐ Reddit (6)
- ☐ YouTube (7)
- ☐ SnapChat (8)
- ☐ TikTok (9)
- ☐ Other (10)
- ☐ I don’t use social media (11)

Q2.6 How often do you use social media?

- ○ Many times, every day (1)
- ○ A few times every day (4)
- ○ About once a day (5)

○ A few times each week (6)

○ About once a week (7)

○ Once or twice a month (8)

○ Less than once a month (9)

○ Never (10)

End of Block: Whatsapp

Start of Block: Mobile Money Survey

Q59 If you want to receive your mobile money payments for the Vaccine Study participation fee with mobile money we need your first name, last name and your mobile money account number.

Q60 Please enter you first name.

____

Q61 Please confirm your first name.

____

Q62 Please enter your last name.

____

Q63 Please confirm your last name.

Q57 In order to receive your payment pay for your participation please enter your mobile money account number.

____

Q58 In order to receive your paymentpay for your participation please reenter your mobile money account number as validation.

____

End of Block: Mobile Money Survey

Start of Block: Block 13

Q67 Please go to a neighbouring house and conduct a spillover interview with the offline spillover survery in your Qualtrics offline application.

End of Block: Block 13

Phase III: Ghana Financial Incentives Spillover Survey

Start of Block: Enumerator survey

Q107 **SPILL-OVER SURVEY -- NEW CONTACTED PARTICIPANTS**

Q2.1 Please enter your device id

____

Q2.2 Please enter your enumerator id

____

Q2.3 Please enter the dwelling sequence number (Number of dwellings you passed by since the previous interview)

____

*

Q2.4 Please enter the village id

____

Q2.5 Did you identify any eligible respondents in this household?

◯ Yes (1)

◯ No (2)

Display This Question:

If Did you identify any eligible respondents in this household? = No

Q3.1 Please Enter the Contact information for this household

◯ There was no answer at this household (1)

◯ There was no one 18 years or over in the household (2)

◯ All household members refused to participate (3)

End of Block: Enumerator survey

Start of Block: Skip Household Questions

Q66 Please register the GPS coordinates on your cell phone.

◯ Longitude (1) ____

◯ Latitude (2) ____

End of Block: Skip Household Questions

Start of Block: Enumerator ID Survey

*

Q4.1 Please describe the random selection of respondent from household members

____

Page Break

Q4.2 Respondent's First name (Respondent's first name as it appears on their Ghana ID Card.)

____

Q4.3 Respondent's Last name (Respondent's last name as it appears on their Ghana ID Card.)

____

End of Block: Enumerator ID Survey

Start of Block: COVID-19 consent

Q1 Your household was selected as one of those to which the survey questions will be asked. You were not selected for any specific reason. Rather, your household was selected randomly from a list of all the households in this village/city. All the information that your household provides is strictly confidential. It will not be shared. Please spare some time to answer the questions. You will receive 5 Cedis for participation in this study. We thank you in advance for your time. Do you consent to take part in the survey?

- ○ Yes, I consent and will take part in the survey. (4)
- ○ No, I do NOT consent and therefore will NOT take part in the survey (5)

End of Block: COVID-19 consent

Start of Block: Consent

Q9.1 We are researchers from the University of Ghana and the University of Oxford, United Kingdom, conducting a study on COVID-19 vaccines. The project is led by Dr. Edward Asiedu of the University of Ghana, working in collaboration with Professor Philip Clarke and Professor Raymond Duch from the University of Oxford, and their team of researchers to better understand vaccine uptake. We very much appreciate your interest in participating in this study. Please carefully consider this information before agreeing to participate by ticking the 'yes' box below. You will be asked some questions about yourself. This should take about 5 minutes. No background knowledge is required. The information that we gather from this study will be used to understand more about COVID-19 vaccinations.

Do I have to take part? Your participation is voluntary. You may withdraw at any point during the questionnaire for any reason before we submit your answers – just indicate to the enumerator that you would like to withdraw. However, we are only able to reimburse participants who complete the full survey (which will include watching a very short video).

How will my data be used? We will not collect any information that would directly identify a participant, so none of the researchers will be able to identify you. The anonymous survey data that you provide will be made available to researchers and may be used in academic publications. Such data will be stored in a secure cloud (eg. OneDrive for Business) of the University of Oxford for a minimum of three years after publication or public release. Anonymous survey data may be securely transferred to, stored and/or processed at a destination outside your country of residence. By completing the interview, you agree to the collection, transfer, storage and processing of your data. This transfer, storage or processing of data will be conducted in such a way that no one will be able to identify you.

Who will have access to my data? The University of Ghana and the University of Oxford will have access to your survey data. The University will process your data for the purpose of the research outlined above. Research is a task that we perform in the public interest. Further information about your rights with respect to your personal data is available from https://compliance.admin.ox.ac.uk/individual-rights. Responsible members of the University of Oxford and funders may be given access to your survey data for monitoring and/or audit of the study to ensure we are complying with guidelines, or as otherwise required by law. Can I opt-out from this panel or ask for my data to be deleted? Participants cannot withdraw themselves or their data once they have submitted the survey responses. We will retain a copy of the data on our, University of Ghana and University of Oxford, servers. This survey is for a research project of the Health Economics Research Centre based at the University of Oxford and the Experimental Development Economics Group at the University of Ghana. The Principal

Researcher is Professor Philip Clarke, who is the Director of the Health Economics Research Centre at the University of Oxford. The Co-Principal Researchers are Dr. Edward Asiedu, who is a development Economist at the University of Ghana and Professor Raymond Duch, who is the Director of the Centre

of Experimental Social Sciences at the University of Oxford. This project has been reviewed by, and received ethics clearance through, the University of Oxford Central University Research Ethics Committee.

Payment. You will receive payment of 5GHS in cash after your participation in today's study is finished. You will be paid immediately. We will follow up and contact you again in 6 weeks – you will receive another 5 GHS when we contact you.

Who do I contact if I have a concern about the study or I wish to complain? If you have a concern about any aspect of this study, please contact Dr. Edward Asiedu at the University of Ghana, email edasiedu@ug.edu.gh and phone at 0550244204 and Professor Raymond Duch at the University of Oxford, email raymond.duch@nuffield.ox.ac.uk and phone number +44 (0)1865 278 515. We will acknowledge your concern within 10 working days and give you an indication of how it will be dealt with. If you remain unhappy or wish to make a formal complaint, please contact the Chair of the Research Ethics Committee at the University of Oxford who will seek to resolve the matter as soon as possible: Medical Sciences Interdivisional Research Ethics Committee (MS IDREC). Research Services, University of Oxford, Wellington Square, Oxford, OX1 2JD Email: ethics@medsci.ox.ac.uk Please note that you may only participate in this survey if you are 18 years of age or over. If you have considered the information we have just presented to you and agree to participate with the understanding that the data (including any personal data) you submit will be processed accordingly, please check the relevant box below to get started.

Q9.2 Please note that you may only participate in this survey if you are **18 years of age or over**.

○ Yes, I certify that I am 18 years of age or over (1)

○ No (2)

Q9.3 If you have read the information above and agree to participate with the understanding that the data (including any personal data) you submit will be processed accordingly, please check the relevant box below to get started.

○ Yes, I agree to take part (1) ○ No (2)

End of Block: Consent

Start of Block: Demographics 1

Q10.1

Thank you for agreeing to participate in our short survey. We would like to ask you some basic questions about yourself. This information is being collected only for statistical purposes. We will ask you a small number of these questions.

Page Break

*

Q10.2 What is your current age?

____

Q10.3 What is your gender?

○ Male (1) ○ Female (2)

○ Other (4) ____

○ Prefer not to say (5)

Page Break

Q10.4 Select the region and district you live in.

Region (1)

District (2)

▼ Ahafo (1) ... Western North ~ Suaman District (276)

Page Break

Q10.5 How many people live in the house together with you (NOT including you) at this moment?

____

Q10.6 How many children below 18 years old are currently living in your home?

____

Q10.7 What is your current working situation?

- ○ Employed (full time) (1)
- ○ Employed (part time) (2)
- ○ Unemployed (3)
- ○ Retired (4)
- ○ Student (5)
- ○ Home maker (6)

Q10.8 How much on average does your household spend in a typical week on food?

____

Q10.9 How much on average does your household spend in a typical week on non-food items (electricity, water, rent, school fees)?

____

Q10.10 How would you rate the overall economic or financial condition of your household today?

- ○ Very good (1)
- ○ Good (2)
- ○ Neither good nor bad (3)
- ○ Bad (4)
- ○ Very bad (5)

Q10.11 What is the highest educational qualification you have completed?

- ○ Never attended (5)
- ○ Nursery (4)
- ○ Kindergarten (9)
- ○ Primary (10)
- ○ JSS/JHS (15)
- ○ Middle (11)
- ○ SSS/SHS (38)
- ○ Vocational/Technical/Commercial (40)

○ Post middle/secondary certificate (41)

○ Post-secondary diploma (42)

○ Bachelor degree (43)

○ Post graduate (Cert. Diploma Masters PHD etc) (45)

End of Block: Demographics 1

Start of Block: Have you received a COVID-19 vaccine?

Q11.1 Have you received at least one shot of a COVID-19 vaccine?

○ Yes, I have received at least one shot a COVID-19 vaccine (1)

○ No, I have not received at least one shot a COVID-19 vaccine (2)

Display This Question:

If Have you received at least one shot of a COVID-19 vaccine? = Yes, I have received at least one shot a COVID-19 vaccine

*

Q11.2 Please enter the date you have received your first COVID-19 vaccine? dd/mm/yyyy

____

Display This Question:

If Have you received at least one shot of a COVID-19 vaccine? = Yes, I have received at least one shot a COVID-19 vaccine

Q11.3 Why did you decide to get vaccinated against COVID-19, what were the reasons? (check all that apply)

- ☐ To protect myself (1)
- ☐ To protect my family (2)
- ☐ To be able to travel (3)
- ☐ To be able to visit family and friends (4)
- ☐ To be able to enter public venues like restaurants, cinemas and concerts (5)
- ☐ To protect the public (people I don't know) (6)
- ☐ Because everyone else will (7)
- ☐ Because friends and family recommend it (8)
- ☐ Because doctors recommend it (9)
- ☐ Because health officials recommend it (10)
- ☐ Because politicians recommend it (11)
- ☐ Because of contact with COVID-19 infected people (12)

☐ Because I had COVID-19 related symptoms (13)

☐ Because it was required for my work or school (14)

☐ Other (15) ____

Display This Question:

If Have you received at least one shot of a COVID-19 vaccine? = Yes, I have received at least one shot a COVID-19 vaccine

Q67 Have you received a second COVID-19 vaccine shot?

○ Yes, I have received a second COVID-19 vaccine (1)

○ No, I have not received a second COVID-19 vaccine (2)

Display This Question:

If Have you received a second COVID-19 vaccine shot? = Yes, I have received a second COVID-19 vaccine

*

Q68 Please enter the date you have received your second COVID-19 vaccine? dd/mm/yyyy

____

Display This Question:

If Have you received at least one shot of a COVID-19 vaccine? = Yes, I have received at least one shot a COVID-19 vaccine

Q105 **FOR ENUMERATOR:** Did you review the repondent's vaccination card?

○ Yes (1)

◯ No (2)

End of Block: Have you received a COVID-19 vaccine?

Start of Block: Spouse and Partner

Q12.1 Do you live with a spouse or partner?

◯ Yes (1)

◯ No (2)

Page Break

Display This Question:

If Do you live with a spouse or partner? = Yes

Q12.2 Did your spouse or partner get a COVID-19 vaccine?

◯ Yes (1)

◯ No (2)

Display This Question:

If Did your spouse or partner get a COVID-19 vaccine? = Yes

*

Q12.3 When did your spouse or partner get a COVID-19 vaccine? dd/mm/yyy

____

Display This Question:

If Did your spouse or partner get a COVID-19 vaccine? = Yes

Q90 **FOR ENUMERATOR:** Did you review the spouse or partner's vaccination card?

◯ Yes (1)

◯ No (2)

End of Block: Spouse and Partner

Start of Block: Reason NOT to GET the COVID-19 vaccine

Display This Question:

If Have you experienced a fever of 100.4F (38C) or greater, a new cough, new loss of taste or smell,... = No

Q13.1 Why did you decide NOT to get vaccinated against COVID-19, what are / were reasons?

(check all that apply)

▢ I don't believe the COVID-19 vaccine will be effective (1)

▢ I was not offered a payment for getting the COVID-19 vaccine (10)

▢ I cannot be vaccinated due to medical reasons as advised by a doctor (2)

▢ I am concerned about dangerous side effects from the COVID-19 vaccine (3)

▢ Enough other people will accept vaccination so I will benefit from herd immunity (4)

▢ I have already been infected with COVID-19 and believe I have developed natural immunity (5)

▢ The COVID-19 virus will not be very harmful to my health (6)

☐ I don't trust the health care providers in this country (7)

☐ I am pregnant or i am about to give birth (9)

☐ I am allergic to the vaccine (11)

☐ I could not afford to take time from my work (12)

☐ Other (8) ____

End of Block: Reason NOT to GET the COVID-19 vaccine

Start of Block: Solar Device

Q14.1 Have you recently purchased a solar charging device since we last spoke to you?

○ Yes, I have recently purchased a solar charging device (1)

○ No, I have not recently purchased a solar charging device (2)

Display This Question:

If Have you recently purchased a solar charging device since we last spoke to you? = Yes, I have recently purchased a solar charging device

*

Q14.2 Please enter the date you have purchased a solar charging device? dd/mm/yyyy

____

End of Block: Solar Device

Start of Block: Network Questions

*

Q15.1 How many villages in the district do you think you have visited in the last month?

____

Q15.2 How many villages in the district do you think you have visited in the last year?

____

Q15.3 Do you have family in other villages in the district?

◯ Yes (1)

◯ No (2)

Display This Question:

If Do you have family in other villages in the district? = Yes

Q15.4 How many family members do you have in another village?

____

Q15.5 Do you have friends and acquaintances who are not part of your family who live in other villages in the district?

◯ Yes (1)

◯ No (2)

Display This Question:

If Do you have friends and acquaintances who are not part of your family who live in other villages... = Yes

Q15.6 How many friends and acquaintances who are not part of your family do you have in another village?

____

End of Block: Network Questions

Start of Block: Network Densitiy Question

Q16.1 How many individuals can you identify in your social network? Think of friends and relatives that live close to you

____

End of Block: Network Densitiy Question

Start of Block: Whatsapp

Q17.1 Do you have WhatsApp?

- ◯ Yes (1)
- ◯ No (2)

Display This Question:

If Do you have WhatsApp? = Yes

Q17.2 How often do you use WhatsApp?

- ◯ Many times, every day (1)
- ◯ A few times every day (4)

About once a day (5)

A few times each week (6)

About once a week (7)

Once or twice a month (8)

Less than once a month (9)

Never (10)

Display This Question:

If Do you have WhatsApp? = Yes

Q17.4 You have WhatsApp groups with...

Close family (parents, children and siblings) (1)

Close friends (4)

College, university or work colleagues (5)

Far family (cousins, uncles, nephews)Neighbors (6)

Church groups (7)

☐ Other groups (8)

☐ I don't have groups (9)

Q17.3 What social media have you used in the last year?

☐ Facebook (1)

☐ Twitter (4)

☐ Instagram (5)

☐ Reddit (6)

☐ YouTube (7)

☐ SnapChat (8)

☐ TikTok (9)

☐ Other (10)

☐ I don’t use social media (11)

Display This Question:

If What social media have you used in the last year? != I don’t use social media

Q17.5 How often do you use social media?

○ Many times, every day (1)

○ A few times every day (4)

○ About once a day (5)

○ A few times each week (6)

○ About once a week (7)

○ Once or twice a month (8)

○ Less than once a month

(9)

○ Never (10)

End of Block: Whatsapp

Start of Block: Network COVID-19 questions

Q69 Thinking back over the past three months have you ever discussed the COVID-19 vaccine with your family members?

○ Yes (1)

○ No (2)

Display This Question:

If Thinking back over the past three months have you ever discussed the COVID-19 vaccine with your f... = Yes

Q70 How often would you say you discussed COVID-19 with your family?

- Every day (1)
- Couple times a week (4)
- At least once a week (5)
- At least once a month (6)

Q71 Thinking back over the past three months have you ever discussed the COVID-19 vaccine with friends and acquaintances in your community?

- Yes (1)
- No (2)

Display This Question:

If Thinking back over the past three months have you ever discussed the COVID-19 vaccine with friend... = Yes

Q73 How often would you say you discussed COVID-19 with friends and acquaintances in your community?

- Every day (1)
- Couple times a week (4)
- At least once a week (5)
- At least once a month (6)

Q74 Thinking back over the past three months have you ever discussed the COVID-19 vaccine with people you know in other villages?

- Yes (1)
- No (2)

Display This Question:

If Thinking back over the past three months have you ever discussed the COVID-19 vaccine with people... = Yes

Q75 How often would you say you discussed COVID-19 with your family?

- Every day (1)
- Couple times a week (4)
- At least once a week (5)
- At least once a month (6)

End of Block: Network COVID-19 questions

Start of Block: COVID-19 Topics

Q76 We are interested in the topics related to COVID-19 that you may have heard about. Could you tell us how much you have heard these COVID-19 topics?

Q77 COVID-19 is a dangerous life-threatening disease. How often have you heard this COVID19 topic?

○ Very Often (1)

○ Often (2)

○ Sometimes (3)

○ Rarely (4)

○ Never (5)

Q78 African communities are concerned that the COVID-19 is not safe for our population. How often have you heard this COVID-19 topic?

○ Very Often (1)

○ Often (2)

○ Sometimes (3)

○ Rarely (4)

○ Never (5)

Q79 In some communities Africans are being paid to get the COVID-19 vaccination. How often have you heard this COVID-19 topic?

○ Very Often (1)

○ Often (2)

○ Sometimes (3)

○ Rarely (4)

Never (5)

Q80 The COVID-19 vaccines that are being given in African communities are not effective – they do not protect us from the COVID-19 virus. How often have you heard this COVID-19 topic?

- Very Often (1)
- Often (2)
- Sometimes (3)
- Rarely (4)
- Never (5)

End of Block: COVID-19 Topics

Start of Block: COVID-19 statements

Q81 We are going to present some statements about the COVID-19 vaccination. Would you tell us how much you agree or disagree with these statements?

Q82 Its each person's responsibility to get the COVID-19 vaccine in order to protect the health of others in our community.

	Strongly Disagree		Indifferent	Strongly Agree	
	0	25	50	75	100
Opinion ()					

Q83 The COVID-19 virus is no longer dangerous for our health.

	Strongly Disagree		Indifferent		Strongly Agree
	0	25	50	75	100
Opinion ()					

Q85 People in our community should get paid in order to encourage them to get the COVID-19 vaccine.

	Strongly Disagree		Indifferent		Strongly Agree
	0	25	50	75	100
Opinion ()					

End of Block: COVID-19 statements

Sampled Village Clusters

Region	Sub.district	Names.of.Community
CENTRAL	OGUAA	ABAMKROM
CENTRAL	APAM	BOMBUA
CENTRAL	OGUAA	ODINA
CENTRAL	Asikuma	MBRAA
CENTRAL	Amanfopong	AMANFOPONG 2
CENTRAL	Odoben	KUNTANASE 2
CENTRAL	Amanfopong	AMANFOPONG 1
CENTRAL	Asikuma	BAAKO
CENTRAL	Amanfopong	MANTE
CENTRAL	wamaso	Wamaso
CENTRAL	Praso	TWANSUKODA
CENTRAL	Praso	Ntafrawaso
CENTRAL	Nyinase	KAYIREKU
CENTRAL	Mokwa	Aboabo
CENTRAL	Bimpong egya	Hasowodze
CENTRAL	Mokwa	moseaso
CENTRAL	KUSHEA	KUSHEA I
CENTRAL	BEDIADUA	BEDIADUA
CENTRAL	AKONFUDI	ENDWA CLINIC
CENTRAL	AKROPONG ODUMASI	AWORABO
CENTRAL	KUSHEA	KUSHEA II
CENTRAL	AKONFUDI	AKONFODI SALEM
CENTRAL	BEDIADUA	ANIAKRAGYA
Akosombo	ABUME	ABUME
AKWAMUFIE APEGUSO	APEGUSO	APEGUSO
ANUM BOSO	ANUM CHPS	Amanfrom
SENCHI	SENCHI FERRY CHPS	ZONGO
AKWAMUFIE APEGUSO	ABOASA	NUDU
SENCHI	NEW AKRADE CHPS	EUROPEAN QUARTERS

CENTRAL	OGUAA	TARKWA SRAHA
CENTRAL	BROFO	MANSO
CENTRAL	APAM	AKYEREMA
CENTRAL	OGUAA	TECHIMAN
CENTRAL	BROFO	SAMPA
CENTRAL	DAGO	ODUMASI
CENTRAL	MUMFORD	AYENSUANO
CENTRAL	OGUAA	MFANTSIMAN
CENTRAL	APAM	AKWAKROM
CENTRAL	APAM	ABUTSIA
CENTRAL	OGUAA	MANKESSIM
CENTRAL	OGUAA	OGUAA
CENTRAL	BROFO	OHUAN
CENTRAL	OGUAA	ESHIEM ABORA
CENTRAL	BROFO	ASEMPANYIN
CENTRAL	OGUAA	TARKWA ODUMASI
CENTRAL	APAM	ZONGO
CENTRAL	OGUAA	TARKWA OSU
CENTRAL	BROFO	ADAM
CENTRAL	BROFO	AJUMAKO ANSA
CENTRAL	DAGO	DAGO BEACH
CENTRAL	OGUAA	ACHIASI
CENTRAL	ONYANDZE	GYANKROM
CENTRAL	MUMFORD	AKYENFO
CENTRAL	BROFO	NGYERESI
CENTRAL	DAGO	DAGO NEWTON
CENTRAL	OGUAA	AKROPONG
CENTRAL	APAM	NKORANSAH
CENTRAL	OSEDZE	OSEDZE
CENTRAL	OGUAA	TARKWA ABOSO
CENTRAL	OSEDZE	FOMENA
CENTRAL	APAM	ABORA
CENTRAL	DAGO	HWIDA

CENTRAL	DAGO	GOMOAMAIN
CENTRAL	DAGO	FAWOMANYE
CENTRAL	ONYANDZE	AMENFI
CENTRAL	APAM	MAMFAM
CENTRAL	BROFO	KYIREN
CENTRAL	OGUAA	ESHIEM MOZANO
CENTRAL	OGUAA	ESHIEM ODUMASI
CENTRAL	MUMFORD	NEW TOWN
CENTRAL	OSEDZE	ABASA
CENTRAL	APAM	KANTAM
CENTRAL	BROFO	ADAA
CENTRAL	OGUAA	DUNKWA
CENTRAL	ONYANDZE	OGUAN
CENTRAL	DAGO	DAGO KOKODO
CENTRAL	OGUAA	AKYIMPIM
CENTRAL	OGUAA	OBOKROM
CENTRAL	DAGO	AMANFUL
CENTRAL	OGUAA	OLEFREKU
CENTRAL	Bedum	MANTSEMANKEBI/DOMEABRA
CENTRAL	Brakwa	ASEREKWA
CENTRAL	Odoben	DOMEABRA
CENTRAL	Anhwiam	AMANBETE
CENTRAL	Brakwa	KWEKUBOA
CENTRAL	Bedum	HABITAT
CENTRAL	Asikuma	OKUKROM
CENTRAL	Amanfopong	AMPAKOBI
CENTRAL	Anhwiam	NKUMKWAA
CENTRAL	Brakwa	BONDAKROM
CENTRAL	Amanfopong	FOSUANSA 2
CENTRAL	Asikuma	ANIEHU
CENTRAL	Asikuma	BENIN
CENTRAL	Amanfopong	FOSUANSA 1
CENTRAL	Odoben	KUNTANASE 3

CENTRAL	Bedum	JUNCTION/ ATU-DAWDA
CENTRAL	Asikuma	ASUOKOR
CENTRAL	Asikuma	KAWANOAPDO
CENTRAL	Odoben	ANIBRINYE
CENTRAL	Brakwa	EDUOSIA
CENTRAL	Asikuma	AGRIC
CENTRAL	Odoben	NKANSAH
CENTRAL	Asikuma	ISAKA EKWRAA
CENTRAL	Asikuma	JAMRA
CENTRAL	Anhwiam	ANHWIAM
CENTRAL	Brakwa	ABRODEASE
CENTRAL	Odoben	ODEBEN 4
CENTRAL	Odoben	ODOBEN 3
CENTRAL	Brakwa	OHENEBRONSO
CENTRAL	Odoben	EKURAKESE
CENTRAL	Asikuma	TOWOBOASE
CENTRAL	Asikuma	BOSOMASE
CENTRAL	Praso	Akwaykom
CENTRAL	Praso	Aklomaim
CENTRAL	Bimpong egya	Caanan
CENTRAL	Nyinase	SUBRISO
CENTRAL	Nyinase	DORKORDOKOR
CENTRAL	Bimpong egya	Pra-Agave
CENTRAL	Praso	DOMEABRA
CENTRAL	Praso	BANKYEASE
CENTRAL	Bimpong egya	Bimpong-agya
CENTRAL	wamaso	Ayaase
CENTRAL	Bimpong egya	Kyeaboso
CENTRAL	Nyinase	KOJOKROM
CENTRAL	Nyinase	OTUKROM
CENTRAL	Bimpong egya	Denyase
CENTRAL	Nyinase	OSENAGYA
CENTRAL	wamaso	Mirekukrom

CENTRAL	Praso	No.1
CENTRAL	Praso	Abodwese
CENTRAL	Praso	BIG BOSS
CENTRAL	Mokwa	Gyaware
CENTRAL	Praso	Mampong
CENTRAL	Praso	Nuamakrom
CENTRAL	Praso	ZONGO
CENTRAL	AKONFUDI	SABINA
CENTRAL	BASOFI NINGO	SEKAMBODUA
CENTRAL	BASOFI NINGO	MPEASEM
CENTRAL	BEREKU	KWAME ANKRA
CENTRAL	BEREKU	MANKENIM
CENTRAL	BEREKU	YAW ATA
CENTRAL	PRASO	PRASO
CENTRAL	BEDIADUA	NTOW KROM
CENTRAL	AKONFUDI	AYITEY
CENTRAL	BEDIADUA	ATWEREBOANDA
CENTRAL	KUSHEA	ARMAH CAMP
CENTRAL	AKONFUDI	GOLD COAST CAMP
CENTRAL	BEDIADUA	ABODWESE
CENTRAL	BEREKU	GYAENKONTABUO
CENTRAL	BEREKU	PRESBY
CENTRAL	BASOFI NINGO	JERUSALEM
CENTRAL	BASOFI NINGO	ASSIN TWEANKA
CENTRAL	BEDIADUA	YAWSAMAN
CENTRAL	BEDIADUA	ANUM
CENTRAL	BEREKU	DANSAME
CENTRAL	BEREKU	MARKET SQUARE
CENTRAL	AKROPONG ODUMASI	NKUKUASA
CENTRAL	BEREKU	DOMINASE II
CENTRAL	BASOFI NINGO	BASOFI-NINGO
CENTRAL	BEREKU	YENTUMINKA
CENTRAL	BEREKU	ATENTAN

CENTRAL	BEDIADUA	ANTOABASA
CENTRAL	AKONFUDI	KEDADWEN
CENTRAL	AKROPONG ODUMASI	KANO
CENTRAL	BASOFI NINGO	BEENUYE
CENTRAL	BEREKU	NINKYISO
CENTRAL	AKONFUDI	ATWEREBOANDA
CENTRAL	PRASO	ASEMPANAYE
CENTRAL	AKROPONG ODUMASI	ODUMASI
CENTRAL	AKONFUDI	AKONFUDI TOWN
CENTRAL	BASOFI NINGO	AMOAKROM
CENTRAL	AKROPONG ODUMASI	KROFOFORODO
CENTRAL	BEREKU	DOMINASE
CENTRAL	BASOFI NINGO	ABEASE
CENTRAL	AKROPONG ODUMASI	WAWASE
CENTRAL	BEDIADUA	KYEIKROM
CENTRAL	KUSHEA	SIMPA
CENTRAL	BASOFI NINGO	NINKYISO
ANUM BOSO	NEW DODI CHPS	New Dodi
AKWAMUFIE APEGUSO	APEGUSO	QUARTERS
Akosombo	NEW COMBINE	CENTRAL COMBINE
ANUM BOSO	DODI ASANTEKROM CHPS	Aberewank)
Adjena Gyakiti	SEDORM CHPS	YANIAMA JUNCTIO
Adjena Gyakiti	ADJENA CHPS	OGLOBO
AKWAMUFIE APEGUSO	FRANKADUA	FRANKADUA
AKWAMUFIE APEGUSO	AKWAMUFIE	AKWAMUFIE /ASAFO
SENCHI	NEW AKRADE CHPS	NEW AKRADE
Adjena Gyakiti	SEDORM CHPS	SEDORM
ANUM BOSO	ANUM CHPS	Ofoase
ANUM BOSO	ANUM CHPS	Christian quarters
ANUM BOSO	DODI ASANTEKROM CHPS	Dodi Asantekrom
ANUM BOSO	ASIKUMA CHPS	Osebeng

Akosombo	PUPUNI	PUPUNI UPPER
ANUM BOSO	BOSO CHPS	Quarters
ATIMPOKU	ATIMPOKU CHPS	AYIMESU
AKWAMUFIE APEGUSO	AYENSU	KORANKYI
Adjena Gyakiti	SEDORM CHPS	ODORNOR
Akosombo	LOWER ASUKWAO	GF
Akosombo	ASUKWAO	WHITE HOUSE
AKWAMUFIE APEGUSO	AYENSU	MPAKADAN QTRS
AKWAMUFIE APEGUSO	MANGOASE	MANGOASE
AKWAMUFIE APEGUSO	AYENSU	ABOMAYAW
SENCHI	SOUTH SENCHI CHPS	ADINAKOPE
AKWAMUFIE APEGUSO	ABOASA	ABOASA
SENCHI	NEW AKRADE CHPS	SENCHI AMANFROM
SENCHI	NEW SENCHI CHPS	APAASO
AKWAMUFIE APEGUSO	FRANKADUA	FINTEY
CENTRAL	Asikuma	KYIRAKAA
CENTRAL	Anhwiam	EDUMANU
CENTRAL	Anhwiam	ANKAASE
CENTRAL	Asikuma	DAM VILLAGE
CENTRAL	Odoben	ODOBEN 1
CENTRAL	Bedum	NWOMASO
CENTRAL	Bedum	AMOANDA
CENTRAL	Brakwa	ANKOBEA/ZONGO
CENTRAL	Odoben	ODOBEN 2
CENTRAL	Odoben	KUNTANASE 1
CENTRAL	Odoben	NANKESE
CENTRAL	Odoben	KWANAN
CENTRAL	Anhwiam	BISEASE
CENTRAL	Odoben	ASEREKWA
CENTRAL	Anhwiam	ESSUMANGWIRA
CENTRAL	Asikuma	EWUSIKWA
CENTRAL	Odoben	OSEI NKWANTA

CENTRAL	Mokwa	Mampoma
CENTRAL	Mokwa	Mokwa
CENTRAL	Mokwa	Kotokye
CENTRAL	Praso	SAKORA PARK
CENTRAL	Bimpong egya	Bimponso
CENTRAL	Bimpong egya	Asamoakrom
CENTRAL	Praso	Ntiamoah
CENTRAL	Nyinase	BONSAHO
CENTRAL	wamaso	Ganukrom
CENTRAL	Praso	AFEDZI
CENTRAL	wamaso	Kwadia
CENTRAL	BEREKU	TWEAPEASE
CENTRAL	AKONFUDI	PEWODIE
CENTRAL	BASOFI NINGO	ZAMBOREE
ANUM BOSO	NEW DODI CHPS	Agu
Akosombo	NEW COMBINE	MANDELA
Adjena Gyakiti	SAPPOR CHPS	NYAMEBENG
ATIMPOKU	TORTIBO CHPS	MANYA
Adjena Gyakiti	ADJENA CHPS	MANYA
AKWAMUFIE APEGUSO	MANGOASE	POWMU
Adjena Gyakiti	ADJENA CHPS	ADJENA
ANUM BOSO	ASIKUMA CHPS	Asikuma
Adjena Gyakiti	GYAKITI CHPS	GYAKITI
AKWAMUFIE APEGUSO	OSIABURA	OSIABURA
ANUM BOSO	ANUM CHPS	Amoanda
SENCHI	NEW SENCHI CHPS	POLICE STATION/ ZONGO
Adjena Gyakiti	SAPPOR CHPS	MARINE
ANUM BOSO	ASIKUMA CHPS	Opokuase
Adjena Gyakiti	KUDIKOPE CHPS	MPAMPROASE
ANUM BOSO	DODI ASANTEKROM CHPS	Mawekope
ATIMPOKU	GHANAKPE CHPS	AKWAMUMAN SCH AREA
Akosombo	ABUME	ATL QUARTER
