# Supplementary material for: Financial incentives for COVID-19 vaccines in a rural low-resource setting: a cluster-randomized trial
Source: Nat Med. 2023 Nov 27;29(12):3193–202. doi: 10.1038/s41591-023-02670-4 (PMC10719106; doi:10.1038/s41591-023-02670-4)
Supplement: Supplementary file 2 — Reporting Summary [file 41591_2023_2670_MOESM2_ESM.pdf]

nature portfolio

Corresponding author(s): Raymond Duch

Last updated by author(s): 2023/26/10

Reporting Summary

Nature Portfolio wishes to improve the reproducibility of the work that we publish. This form provides structure for consistency and transparency in reporting. For further information on Nature Portfolio policies, see our Editorial Policies and the Editorial Policy Checklist.

Statistics

For all statistical analyses, confirm that the following items are present in the figure legend, table legend, main text, or Methods section.

n/a	Confirmed	
☐	☒	The exact sample size (*n*) for each experimental group/condition, given as a discrete number and unit of measurement
☐	☒	A statement on whether measurements were taken from distinct samples or whether the same sample was measured repeatedly
☐	☒	The statistical test(s) used AND whether they are one- or two-sided<br>*Only common tests should be described solely by name; describe more complex techniques in the Methods section.*
☐	☒	A description of all covariates tested
☐	☒	A description of any assumptions or corrections, such as tests of normality and adjustment for multiple comparisons
☐	☒	A full description of the statistical parameters including central tendency (e.g. means) or other basic estimates (e.g. regression coefficient) AND variation (e.g. standard deviation) or associated estimates of uncertainty (e.g. confidence intervals)
☐	☒	For null hypothesis testing, the test statistic (e.g. *F*, *t*, *r*) with confidence intervals, effect sizes, degrees of freedom and *P* value noted<br>*Give P values as exact values whenever suitable.*
☐	☒	For Bayesian analysis, information on the choice of priors and Markov chain Monte Carlo settings
☒	☐	For hierarchical and complex designs, identification of the appropriate level for tests and full reporting of outcomes
☒	☐	Estimates of effect sizes (e.g. Cohen's *d*, Pearson's *r*), indicating how they were calculated

Our web collection on statistics for biologists contains articles on many of the points above.

Software and code

Policy information about availability of computer code

Data collection	Data collection was conducted using Qualtrics First Release 2005.
Data analysis	Analyses were performed using R version 4.2.0, including the following packages (versions): htmlTable, stargazer, data.table, DescTools, ggpubr, stats (4.0.3), tidyverse (1.3.0), estimatr (0.28.0), readr (1.4.0), dplyr (1.0.5), lubridate (1.7.10), hdm (0.3.1), car (3.0.10), MASS (7.3.53), sandwich (3.0.0), misty (0.4.6), foreign (0.8.80), readxl (1.3.1), mlogit (1.1-1), nnet (7.3), aod (1.3.2), RVAideMemoire (0.981-2) and quantreg (5.75) cobat (4.4.1).

For manuscripts utilizing custom algorithms or software that are central to the research but not yet described in published literature, software must be made available to editors and reviewers. We strongly encourage code deposition in a community repository (e.g. GitHub). See the Nature Portfolio guidelines for submitting code & software for further information.

Data

Policy information about availability of data

All manuscripts must include a data availability statement. This statement should provide the following information, where applicable:

- Accession codes, unique identifiers, or web links for publicly available datasets
- A description of any restrictions on data availability
- For clinical datasets or third party data, please ensure that the statement adheres to our policy

All anonymized data used in the analysis are publicly available to anyone at \url{https://doi.org/10.7910/DVN/X5MBHP}. All data are shared in a public registry

\url{https://doi.org/10.7910/DVN/X5MBHP}. The data are freely accessible \url{https://doi.org/10.7910/DVN/X5MBHP}. Source data are provided with this paper.

\section{Code Availability} All codes are available at \url{https://doi.org/10.7910/DVN/X5MBHP}. The code is freely accessible at \url{https://doi.org/10.7910/DVN/X5MBHP}. Source data are provided with this paper.

Research involving human participants, their data, or biological material

Policy information about studies with human participants or human data. See also policy information about sex, gender (identity/presentation), and sexual orientation and race, ethnicity and racism.

Reporting on sex and gender	The results are reported with a breakdown by gender -- gender is a self-reported category including male, female, and other.
Reporting on race, ethnicity, or other socially relevant groupings	Results are analyzed by other groupings, including: age, mean food expenditures, mean other household expenditures, education and social media usage.
Population characteristics	See above.
Recruitment	Village clusters were randomly selected from a comprehensive list of villages in each of the six districts. Individual households within villages were randomly selected using a version of the Kish random walk methodology.
Ethics oversight	The design was approved by the Economics departmental research ethics committee (DREC) of the Social Science Division of the University of Oxford with the University of Ghana recognizing the ethics oversight of the University of Oxford DREC.

Note that full information on the approval of the study protocol must also be provided in the manuscript.

Field-specific reporting

Please select the one below that is the best fit for your research. If you are not sure, read the appropriate sections before making your selection.

☐ Life sciences ☒ Behavioural & social sciences ☐ Ecological, evolutionary & environmental sciences

For a reference copy of the document with all sections, see nature.com/documents/nr-reporting-summary-flat.pdf

Behavioural & social sciences study design

All studies must disclose on these points even when the disclosure is negative.

Study description	We implemented a clustered randomized controlled trial with 7,227 residents in six rural Ghana Districts to determine whether financial incentives produce substantial increases in COVID-19 vaccine uptake. This is quantitative study.
Research sample	7,227 residents in six rural Ghana Districts
Sampling strategy	In each of the six Districts, our District Health Office partners identified villages that could be feasibly enumerated (the primary consideration here was either road access or the quality of the road access); villages are ranked according to their population size; each four consecutive villages are designated a quadruplet. A typical district has approximately 50 quadruplets. In each District we randomly select 13 quadruplets with probabilities weighted by the quadruplet's share of the total population of the villages being considered in the District. Within each quadruplet, villages are randomly assigned to receive one of four video treatment arms: a placebo, a standard health message, a high cash incentive (60 Ghana Cedis/\$10) and a low cash incentive (20 Ghana Cedis/\$3). A total of 323 villages were included with about 80 villages assigned to each of the four treatment arms. Within each village we randomly selected 21 households.
Data collection	The experiment has four treatments that are delivered in a short video:<br>Treatment 1: A 45-second placebo video that provides general information about the benefit of using solar power to charge household electrical appliances.<br>Treatment 2: A 45-second standard health COVID-19 vaccine promotional and information video (modeled on the videos produced by the U.S. Center for Disease Control).<br>Treatment 3: Low Cash Incentive treatment -- the first 30 seconds are identical to the Health video -- the last 15 seconds inform viewers that that they will earn $3 (20 Ghana Cedis) if they receive the COVID-19 vaccine within the next 6 weeks.<br>Treatment 4: High Cash Incentive treatment -- the first 30 seconds are identical to the Health video -- the last 15 seconds inform viewers that that they will earn $10 (60 Ghana Cedis) if they receive the COVID-19 vaccine within the next 6 weeks.<br><br>The four treatment videos are available at \\ \url{https://www.youtube.com/watch?v=PeM1cpCU0bA&list=PLBGbIwQfB9sez5Ww6xcKmwBQa45Z3Vvwd}. Subjects viewed the videos on a tablet that was presented to them by an enumerator. The randomizaton of video presentations was managed via a downloaded Qualtrics script from the Candour server. The interventions were conducted in person by enumerators from the University of Ghana. Subjects were compensated with 5 Ghana Cedis immediately after this Phase I intervention; they received an additional 5 Ghana Cedis for their participation in the Phase II/III post-treatment survey. Signed consent to participate in the RCT was obtained from each participants -- the full text of the

	signed consent can be found in the text of the Phase I questionnaire.
Timing	The trial interventions began on February 5 2022. Beginning on April 13, 2022, two months after the initial intervention, subjects were contacted via telephone and asked a short survey including a question about their vaccination status (the survey is available in the Online Supplementary Materials). In this Phase II, a total of 2,158 subjects, from the 6,126 originally treated subjects, were successfully contacted by telephone (a response rate of approximately 35%).<br>Phase III began on June 15, 2022 with two components. In the Verified Vaccine Status component, enumerators contacted all subjects who were not successfully contacted via telephone in Phase II of the trial. These subjects were asked an identical set of questions to those included in the Phase II post-treatment survey. A second component of Phase III entailed an Enumeration of a Sample of Spillover Households. This Spillover Sample consisted of non-treated subjects in treated villages. Finally, between October 15 and November 30, 2022, Phase IV, Verified Vaccine Status, entailed the official verification of the vaccine status of all subjects.
Data exclusions	In the last stage of the project that involved collection of verified vaccination status we were unable to get reliable data from two of the six districts.
Non-participation	In the post-treatment phase we were unable to contact 1,878 of the 6,126 subjects who participated in the Baseline Treatment Intevention phase.
Randomization	Village clusters were randomly selected from a comprehensive list of villages in each of the six districts. Individual households within villages were randomly selected using a version of the Kish random walk methodology.

Reporting for specific materials, systems and methods

We require information from authors about some types of materials, experimental systems and methods used in many studies. Here, indicate whether each material, system or method listed is relevant to your study. If you are not sure if a list item applies to your research, read the appropriate section before selecting a response.

Materials & experimental systems

n/a	Involved in the study
☒	☐ Antibodies
☒	☐ Eukaryotic cell lines
☒	☐ Palaeontology and archaeology
☒	☐ Animals and other organisms
☒	☐ Clinical data
☒	☐ Dual use research of concern
☒	☐ Plants

Methods

n/a	Involved in the study
☒	☐ ChIP-seq
☒	☐ Flow cytometry
☒	☐ MRI-based neuroimaging
